# Morphological and evolutionary insights into the keystone element of the human foot’s medial longitudinal arch

**DOI:** 10.1038/s42003-023-05431-8

**Published:** 2023-10-19

**Authors:** Rita Sorrentino, Kristian J. Carlson, Caley M. Orr, Annalisa Pietrobelli, Carla Figus, Shuyuan Li, Michele Conconi, Nicola Sancisi, Claudio Belvedere, Mingjie Zhu, Luca Fiorenza, Jean-Jacques Hublin, Tea Jashashvili, Mario Novak, Biren A. Patel, Thomas C. Prang, Scott A. Williams, Jaap P. P. Saers, Jay T. Stock, Timothy Ryan, Mark Myerson, Alberto Leardini, Jeremy DeSilva, Damiano Marchi, Maria Giovanna Belcastro, Stefano Benazzi

**Affiliations:** 1 Department of Biological, Geological and Environmental Sciences, University of Bologna, Bologna, 40126 Italy; 2 Department of Integrative Anatomical Sciences, Keck School of Medicine, University of Southern California, Los Angeles, 90033 USA; 3 Evolutionary Studies Institute, University of the Witwatersrand, Johannesburg, WITS 2050 South Africa; 4 Department of Cell and Developmental Biology, University of Colorado School of Medicine, Aurora, CO 80045 USA; 5 Department of Anthropology, University of Colorado Denver, Denver, CO 80217 USA; 6 Department of Cultural Heritage, University of Bologna, Ravenna, 48121 Italy; 7 Department of Orthopaedic Surgery, University of Colorado, Denver, CO USA; 8 Department of Industrial Engineering, Health Sciences and Technologies, Interdepartmental Centre for Industrial Research (HST–ICIR), University of Bologna, Bologna, 40136 Italy; 9 Laboratory of Movement Analysis and Functional Evaluation of Prostheses, IRCCS Istituto Ortopedico Rizzoli, Bologna, Italy; 10 Monash Biomedicine Discovery Institute, Department of Anatomy and Developmental Biology, Monash University, Melbourne, VIC 3800 Australia; 11 Chaire Internationale de Paléoanthropologie, CIRB (UMR 7241–U1050), Collège de France, Paris, France; 12 Max Planck Institute for Evolutionary Anthropology, Leipzig, 04103 Germany; 13 Department of Geology and Paleontology, Georgian National Museum, Tbilisi, 0105 Georgia; 14 Centre for Applied Bioanthropology, Institute for Anthropological Research, Zagreb, 10000 Croatia; 15 Human and Evolutionary Biology Section, Department of Biological Sciences, University of Southern California, Los Angeles, 90089 USA; 16 Department of Anthropology, Washington University in St. Louis, St. Louis, MO 63130 USA; 17 Center for the Study of Human Origins, Department of Anthropology, New York University, New York, 10003 USA; 18 Centre for the Exploration of the Deep Human Journey, University of the Witwatersrand, Johannesburg, Wits 2050 South Africa; 19 Naturalis Biodiversity Center, 2333 CR Leiden, the Netherlands; 20 Department of Anthropology, Western University, London, Ontario N6A 3K7 Canada; 21 Department of Anthropology, The Pennsylvania State University, State College, PA 16802 USA; 22 Department of Anthropology, Dartmouth College, Hanover, NH 03755 USA; 23 Department of Biology, University of Pisa, Pisa, 56126 Italy

**Keywords:** Biological anthropology, Biodiversity, Bone, Archaeology

## Abstract

The evolution of the medial longitudinal arch (MLA) is one of the most impactful adaptations in the hominin foot that emerged with bipedalism. When and how it evolved in the human lineage is still unresolved. Complicating the issue, clinical definitions of flatfoot in living *Homo sapiens* have not reached a consensus. Here we digitally investigate the navicular morphology of *H. sapiens* (living, archaeological, and fossil), great apes, and fossil hominins and its correlation with the MLA. A distinctive navicular shape characterises living *H. sapiens* with adult acquired flexible flatfoot, while the congenital flexible flatfoot exhibits a ‘normal’ navicular shape. All *H. sapiens* groups differentiate from great apes independently from variations in the MLA, likely because of bipedalism. Most australopith, *H. naledi*, and *H. floresiensis* navicular shapes are closer to those of great apes, which is inconsistent with a human-like MLA and instead might suggest a certain degree of arboreality. Navicular shape of OH 8 and fossil *H. sapiens* falls within the normal living *H. sapiens* spectrum of variation of the MLA (including congenital flexible flatfoot and individuals with a well-developed MLA). At the same time, *H. neanderthalensis* seem to be characterised by a different expression of the MLA.

## Abstract (web summary)

A GM study diachronically examined evolution of the medial longitudinal arch and variation as expressed by the navicular bone, identifying navicular traits associated with particular locomotor behaviors, subsistence strategies, and foot types

## Introduction

**Fig. 1:**
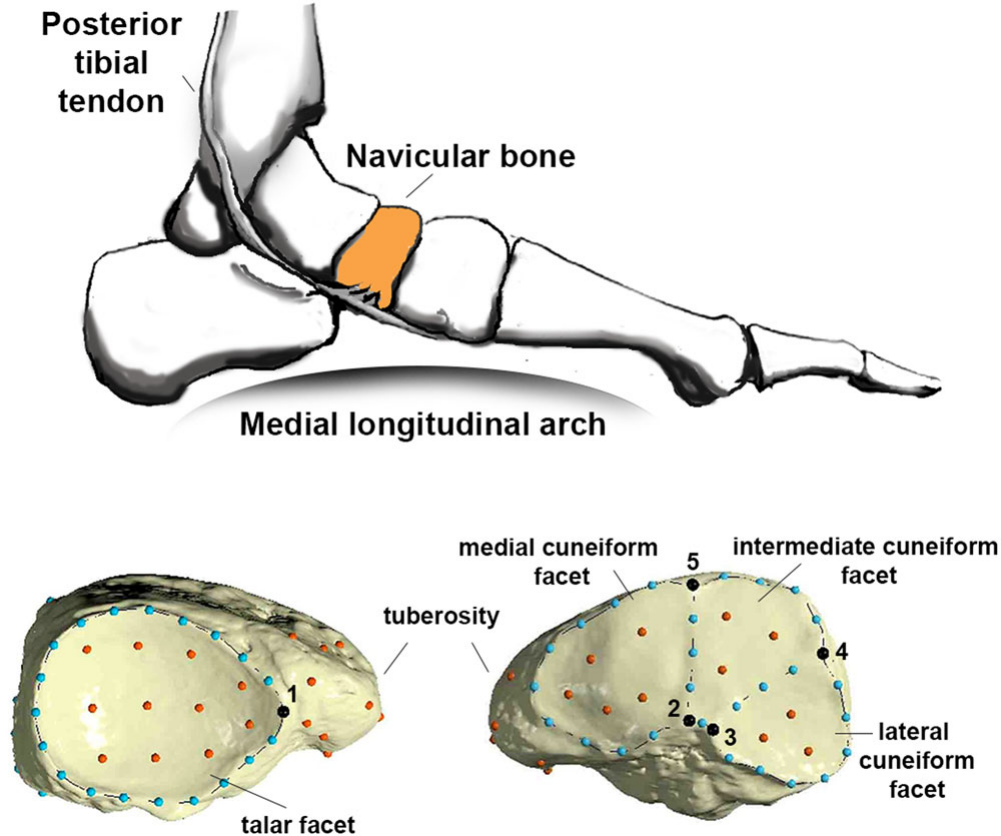
Anatomical position of the navicular (orange) in the medial column of the foot (top). Along the bottom, renderings of an archaeological *H. sapiens* navicular (from the Frassetto identified human skeletal collection – University of Bologna) are illustrated in proximal (bottom left) and distal (bottom right) views. Placement of landmark and semi-landmark configurations are shown: five fixed landmarks (black), 46 curved semi-landmarks (light blue) describing corresponding articular surface contours, and 34 surface semi-landmarks (orange) on articular surfaces and the navicular tuberosity.

The navicular (Fig. 1) is one of seven tarsal bones and contributes to the medial column of the foot. The navicular articulates with the talus proximally, three cuneiforms distally, and the tuberosity on its medial surface serves as the attachment for *m. tibialis posterior*. It rarely articulates with the cuboid in *Homo sapiens*, but often does so in other great apes^1^. The navicular is the keystone of the *H. sapiens* medial longitudinal arch (MLA) thanks to its position in the midfoot. The MLA, together with the lateral longitudinal arch, form the unique longitudinal arch (LA) of the *H. sapiens* foot. These arches are also supported by the transverse (mediolateral) arch in the *H. sapiens* foot^2,3^, which is expressed to varying degrees in other primates, whereas only *H. sapiens* express a LA in high frequency^4^.

Functionally, during walking and running the LA transforms the *H. sapiens* foot from a deformable construct at heel strike to a stiff lever at push-off, thus promoting the storage and release of elastic potential energy during the stance phase via the stretch of the plantar aponeurosis and other plantar soft tissues, such as the calcaneonavicular (spring) ligament^5–7^. This energy-saving mechanism is considered an advantageous adaptation for bipedality by increasing efficiency for long-distance walking and running^8,9^. Ankle and foot bone morphologies have shown a correlation with LA variation (e.g., pes cavus, pes planus, and normal feet) in living *H. sapiens*^10–13^. A combination of the geometry of the ankle and foot bones, ligaments, plantar aponeurosis, and muscle-tendon complexes contributes to the complicated structure of the LA^14^. However, the exact role of each component is still debated^15^.

The LA distinguishes the *H. sapiens* foot from that of (non-human) great apes^4^. The earliest appearance of the LA in the human fossil records remains controversial since it is still debated whether this derived characteristic arose within *Australopithecus* or *Homo*^13,16–19^. Aside from preserved footprints, the only evidence attesting to the evolution of the LA of the foot consists of hominid pedal bony indicators. Because flatness of the MLA is considered the primary indicator of flatfoot in living *H. sapiens*^20^ and great apes^21^, and given the pivotal role of the navicular in determining form of the MLA, morphological differences between naviculars of great apes and archaeological *H. sapiens* have been used to infer expression of LA form in fossil hominins^22–26^. Such studies have promoted binary interpretations in which hominins with a navicular similar to those of great apes have been attributed an absent MLA, while hominins with a navicular similar to those of archaeological *H. sapiens* have been attributed a MLA. Considering that *H. sapiens* from archaeological contexts or from museum collections are usually without any accompanying diagnostic information on foot type, it cannot be truly known whether archaeological *H. sapiens* possessed a well-developed MLA during life. In other words, a correlation between archaeological *H. sapiens* navicular form and the presence of a well-developed MLA typically has been assumed.

In living *H. sapiens*, clinical data have attested that the MLA is absent in infants and typically develops during childhood (7–10 years); however, should the MLA fail to form, children will retain a flatfoot form^27,28^. In living *H. sapiens*, “flatfoot” may present as one of multiple conditions (rigid or flexible flatfoot) that either develop in childhood (congenital) or that develop secondarily during adulthood (acquired). One particular form of flatfoot (i.e., congenital flexible flatfoot) is debated whether it can be considered as a deformity or a disease because it is associated with a normal (although low) MLA when the foot is not loaded, and it is generally pain-free during normal walking^20^. Additionally, variation in MLA height is also assumed to be related to footwear, lifestyles and subsistence strategies. For example, most barefoot hunter-gatherers show wider and flatter feet compared to shod populations^29,30^. However, conflicting evidence exists regarding an increased foot width in barefoot populations, and only a few studies have controlled for confounding variables (e.g., sex, ethnicity, BMI)^31,32^.

Considering the complex structural basis of the MLA and flatfoot condition^33^, this study aims to evaluate hominid navicular morphology and to assess its anatomical correlates with the MLA (Fig. 1). First, we investigate living *H. sapiens* that have been clinically diagnosed as having a well-developed MLA (i.e., high arch but pes cavus excluded) or flatfooted feet and test whether navicular morphology differs between them. The flatfoot sample includes individuals exhibiting either congenital flexible flatfoot or adult acquired flexible flatfoot. Our objective is to investigate the impact of congenital flexible flatfoot on diversity in modern human navicular morphology, considering the ongoing debate surrounding its classification as a disease. We hypothesize that this condition may have originated from a neutral flatfoot in our ancestors and was subsequently maintained in *H. sapiens*. The study excludes individuals with rigid flatfoot, as it is associated with tarsal coalition^28^, a condition presumed to not be present in fossil hominins or the *H. sapiens* investigated here. Second, we compare great apes with different groups of living, archaeological, and fossil *H. sapiens* to identify navicular traits associated with particular locomotor behaviors, subsistence strategies, and foot types. Finally, we assess fossil hominin naviculars for an exclusive set of ape-like features, human-like features, including those corresponding to different living *H. sapiens* foot types (e.g., flatfoot vs. arched foot), or for a mix of features. Particular attention was devoted to the OH 8 navicular, as its morphological affinity with *H. sapiens* and taxonomic allocation to *H. habilis* or *P. boisei* have been questioned^25,34–36^.

## Results

### Living *H. sapiens*: flatfeet vs control groups

**Fig. 2:**
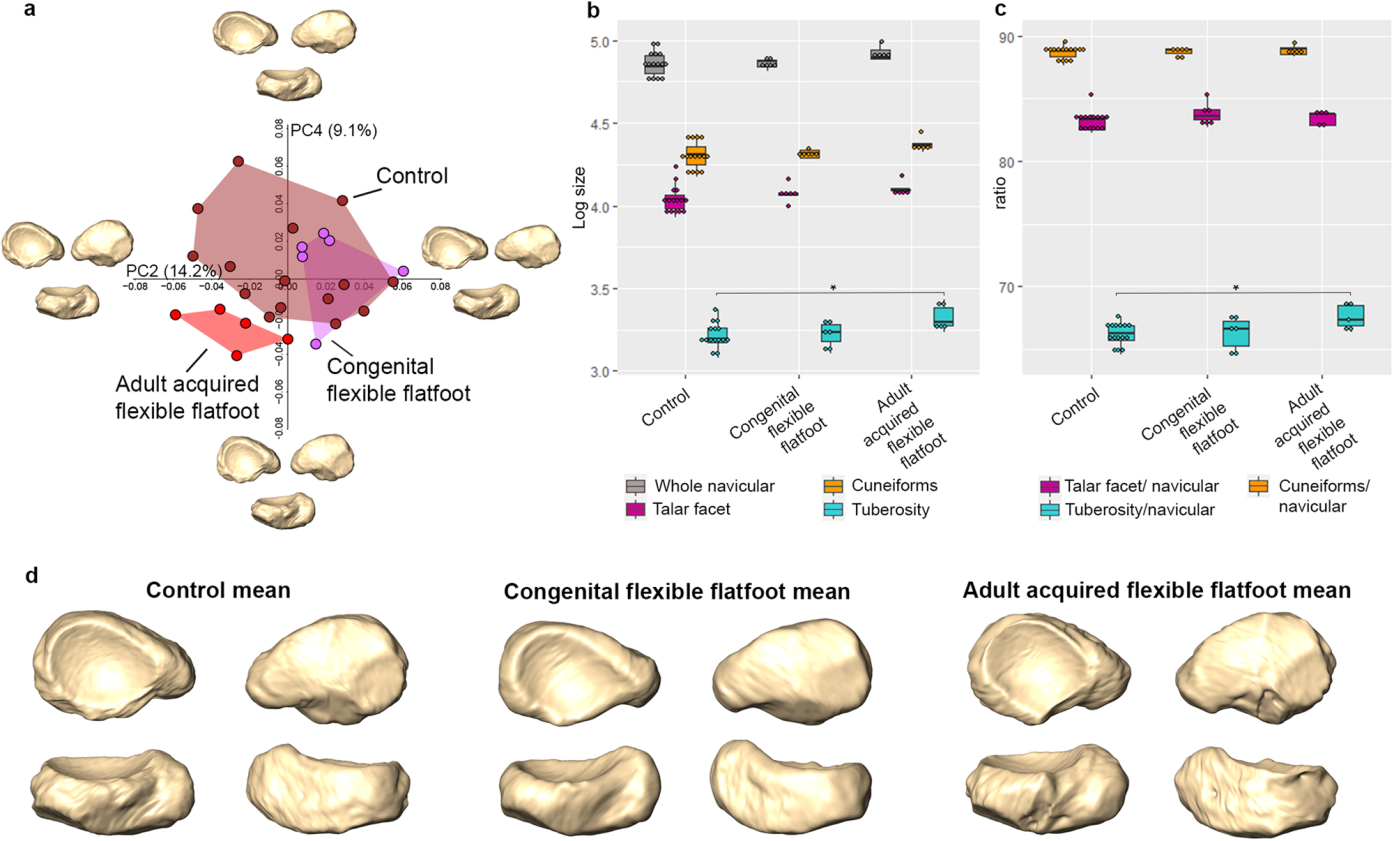
PCA plot showing the PCs (i.e., PC2 and PC4) that detected differences among naviculars of individuals diagnosed with congenital flexible flatfoot, adult acquired flexible flatfoot and a normal or control condition. Extreme shapes along the PCs are illustrated in proximal (top left), distal (top right), and plantar (bottom) views, respectively (**a**). Box plot representing size distribution (lnCS) of the overall navicular, talar facet, cuneiform facets, and tuberosity (**b**), as well as the ratio between lnCS of the overall navicular and lnCS of the talar facet, cuneiform facets, and tuberosity, respectively (**c**), of congenital flexible flatfoot (*n* = 6 biologically independent samples), adult acquired flexible flatfoot (*n* = 5 biologically independent samples) and a normal or control condition (*n* = 15 biologically independent samples) groups. Asterisks mark comparisons in which null hypotheses of no difference could be rejected using Kruskal-Wallis or ANOVA (*p* = <0.05). Box components include the median (the horizontal bar), the upper and lower quartiles (limits of the boxes), and the extremes of each range (terminus of whiskers). Group mean configurations (control, congenital and adult acquired flexible flatfoot, respectively) are visualised in proximal (top left), distal (top right), plantar (bottom left), and dorsal (bottom right) views (**d**).

A principal component analysis (PCA) of Procrustes shape coordinates was conducted to explore morphological differences among living *H. sapiens* individuals diagnosed with congenital flexible flatfoot, adult acquired flexible flatfoot, and a control sample composed of individuals who exhibit a clinically verified well-developed longitudinal arch (Figs. 2a, S1). Permutation tests on the first six principal components (PCs; all contributing to >5% of variance) cumulatively explaining about 71% of the total variance in the sample identify significant differences between the adult acquired flexible flatfoot group versus the congenital flexible flatfoot and control groups along PC2 and PC4, whereas the congenital flexible flatfoot group differs from the control group along PC6 (*p* = 0.039, Table S1). Shape differences were not significantly associated with size (natural logarithm of centroid size; lnCS) along the first six PCs. In contrast, we observed differences in lnCS between the control and adult acquired flexible flatfeet for the tuberosity (i.e., larger in the latter; Fig. 2b) and for the ratio between tuberosity size and overall navicular size (Fig. 2c). Individuals with adult acquired flexible flatfoot exhibit an approximately 2% increase in the ratio of tuberosity size to overall navicular size compared to the controls (Table S2 and Fig. 2b, c). This result is also qualitatively visible when comparing surfaces of group mean renderings (Fig. 2d, plantar view). Both congenital and adult acquired flexible flatfeet are characterised by a similarly larger (but statistically non-significant) talar facet associated with an extended tuberosity compared to the control group (Table S2 and Fig. 2d). In addition, a proximodistally elongated plantar aspect (Fig. S2) and a greater distal convexity of the navicular characterize the adult acquired flexible flatfoot group compared to other groups (Figs. 2d, S2).

### Comparisons of great apes, *H. sapiens*, and fossil hominins

A Procrustes ANOVA indicates that genus explained approximately 52% of the variance in navicular shape (R^2^ = 0.52), whereas lnCS and the genus:lnCS interaction term explained only 0.2% and 0.8%, respectively, of the variance in overall navicular shape (Table 1). Thus, pairwise comparisons of navicular shape reveal that the null hypothesis of no difference can be rejected for all extant taxa (*p* = 0.001). The phylogenetic signal on navicular shape variation among extant taxa is low (K = 0.2412, *p* = 0.001) indicating that extant taxa are less similar to one another in navicular shape than expected under a Brownian motion evolutionary model. Low K values could suggest that other factors, such as adaptation, produced departures from a purely Brownian motion signal.

**Table 1:** Procrustes ANOVA testing differences among extant genera (great apes and *H. sapiens*, fossil hominins excluded).

Variables	Df	SS	MS	R^2^	F	Z	*p*-value
Genus	3	2.5980	0.86601	0.52027	125.6836	6.9012	0.001^a^
lnCS	1	0.0130	0.01304	0.00261	1.8931	2.0519	0.024^a^
Genus:lnCS	3	0.0398	0.01327	0.00797	1.9256	3.4765	0.001^a^
Residuals	340	2.3427	0.00689	0.46915			
Total	347	4.9936					

*Df* degree of freedom, *SS* Procrustes distance sum of squares, *MS* mean squares distance, *R*^*2*^ coefficient of determination, F = effect type; Z = effect size; *lnCS* natural logarithm of centroid size.

^a^Significant *p*-value (*p* < 0.05).

**Fig. 3:**
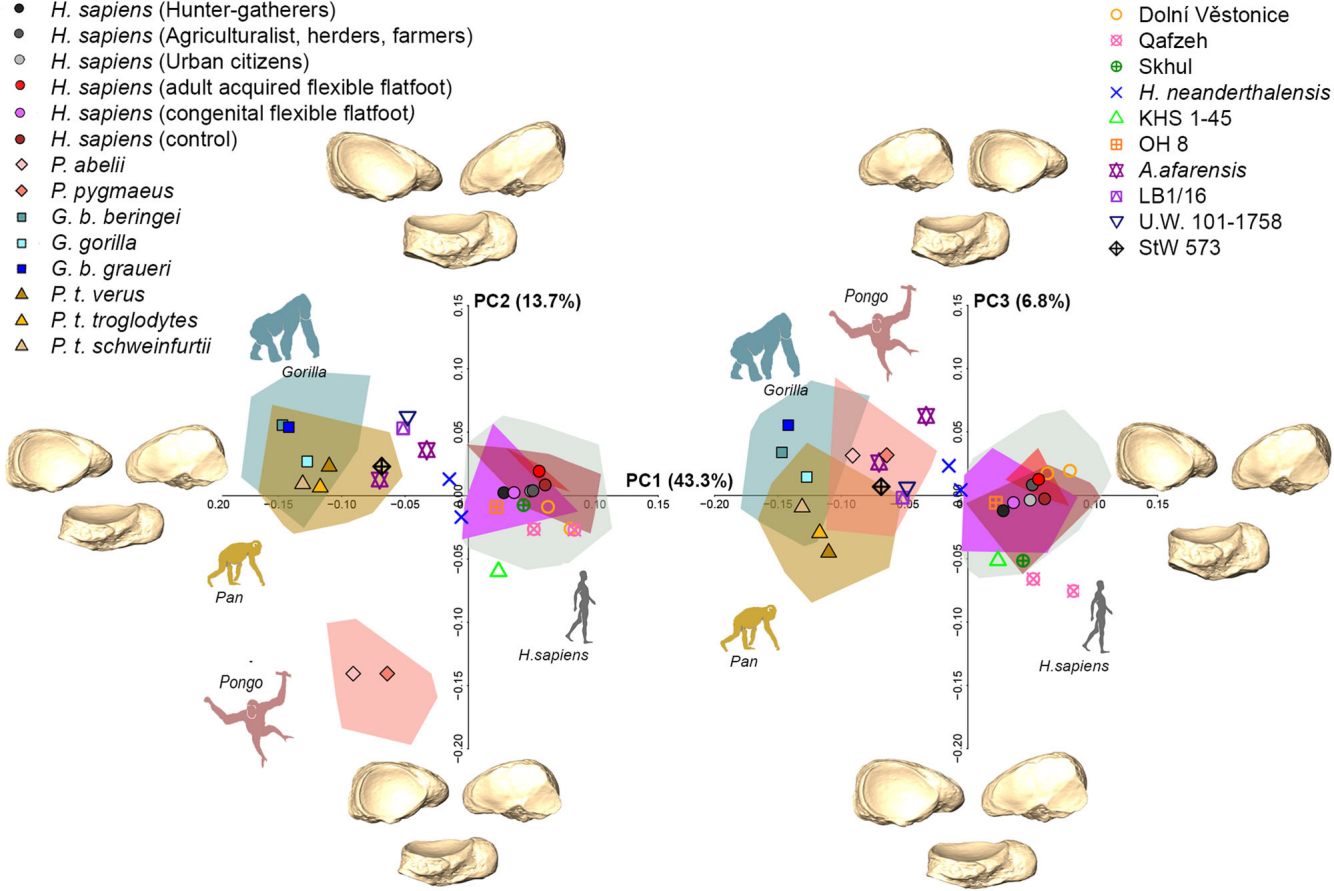
PCA plots of great apes, *H. sapiens*, and fossil hominins. PCA plots showing PC1 vs. PC2 (on the left) and PC1 vs. PC3 (on the right). Each group’s mean is shown together with individual fossil hominins (see SI Fig. S3 for comparison). Navicular shape differences along the first three PCs are represented by the respective renderings in proximal (top left), distal (top right), and plantar (bottom) views.

Figure 3 shows the shape-space PCA of the entire sample (see also Fig. S3). The first three PCs account for 63.8% of the total variance, and all of them are correlated with size (Pearson test; PC1: r = 0.201, *p* < 0.001; PC2: r = 0.350, *p* < 0.001; PC3: r = 0.190, *p* = 0.001), ultimately suggesting that a static allometric component drives some of the observed shape differences. In particular, PC1 (43.3%) segregates the combined living and archaeological *H. sapiens* sample from the great apes (*p* = 0.001), with *H. sapiens* (positive scores) showing an anteroposteriorly broad navicular with mediolaterally reduced length relative to great apes (negative scores; Fig. 3; Table S3). Interestingly, though the morphological variability of the great ape sample effectively conceals intraspecific variability within the *H. sapiens* sample, hunter-gatherer *H. sapiens* still show distinctly lower PC1 scores along with individuals characterised by congenital flexible flatfeet. PC2 (13.7%) most clearly separates *Pongo* from African great apes and *H. sapiens* (*p* = 0.001), as well as *Gorilla* from *H. sapiens* (*p* = 0.001) and *Pan* (*p* = 0.006). The latter difference seems likely driven by the two *Gorilla beringei* groups that plot along more positive PC2 scores diverging from *Pan* and *H. sapiens* means, whereas the *Gorilla gorilla* group mean is very close to PC2 scores also covered by *Pan* and *H. sapiens* means. Positive scores along PC2 (i.e., African great apes and *H. sapiens*) account for an overall larger navicular, with a more elongated mediolateral length and a more pronounced and plantarly oriented tuberosity, a more rounded dorsolateral corner with a more oblique rather than vertical lateral margin, a more obliquely oriented long axis of the talar facet, and a more elliptical shaped talar facet with respect to negative PC2 scores. PC3 (6.8%) separates all taxa (*p* = 0.001) except *Gorilla* from *Pongo* (Table S3), with negative PC3 scores showing a greater mediolateral length and a decreased anteroposterior width compared to positive PC3 scores. Unlike PC2, the positive scores of both PC1 and PC3 seem to show a more circular shaped talar facet (Fig. 3).

**Fig. 4:**
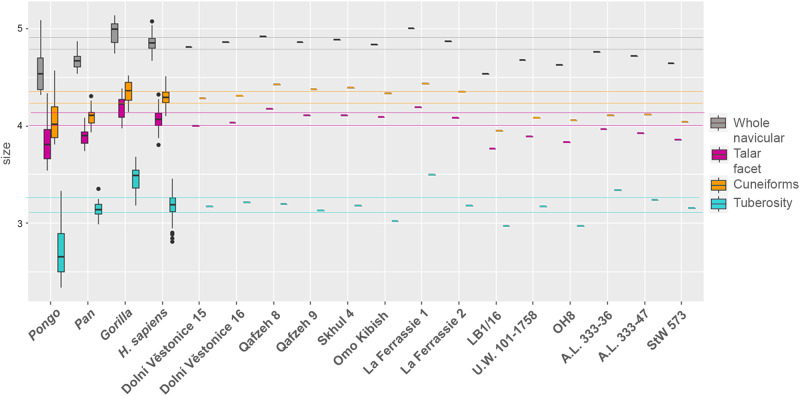
Box plot representing size distribution (lnCS) of the overall navicular, talar facet, cuneiform facets, and tuberosity. The sample represented in the box plot consists of *Pongo* (*n* = 21 biologically independent samples), *Pan* (*n* = 46 biologically independent samples), *Gorilla* (*n* = 35 biologically independent samples), *Homo sapiens* (i.e., combined living and archaeological *H. sapiens;* *n* = 246 biologically independent samples), and fossil hominins (*n* = 14 biologically independent samples). Box components include the median (the horizontal bar), the upper and lower quartiles (limits of the boxes), the extremes of each range (terminus of whiskers), and the black dots are outliers. To facilitate comparisons with the combined living and archeological *H. sapiens* group, color-coded horizontal lines bracket the first quartile to the third quartile of variation within combined living and archaeological *H. sapiens*.

**Fig. 5:**
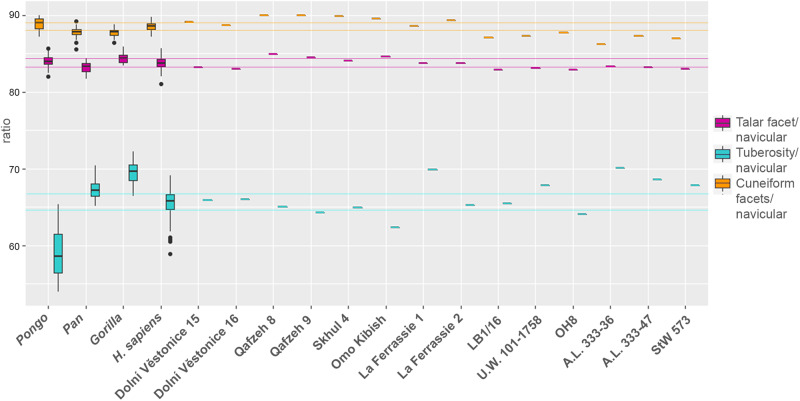
Box plot representing ratios between the overall navicular lnCS and the lnCS of the talar facet, cuneiform facets and the tuberosity of the overall sample, respectively. The sample represented in the box plot consists of *Pongo* (*n* = 21 biologically independent samples), *Pan* (*n* = 46 biologically independent samples), *Gorilla* (*n* = 35 biologically independent samples), *Homo sapiens* (i.e., combined living and archaeological *H. sapiens;* *n* = 246 biologically independent samples), and fossil hominins (*n* = 14 biologically independent samples). Box components include the median (the horizontal bar), the upper and lower quartiles (limits of the boxes), the extremes of each range (terminus of whiskers), and the black dots are outliers. To facilitate comparisons with the combined living and archaeological *H. sapiens* group, color-coded horizontal lines bracket the first quartile to the third quartile of variation within combined living and archaeological *H. sapiens*.

Based on a partial least squares analysis, it was observed that an increment in navicular size corresponds to increased anteroposterior width of the navicular and reduced mediolateral navicular length in hominids (Fig. S4). Differences in lnCS distinguish all taxa except for *Pan* and *Pongo*, which are not differentiated in the overall navicular, nor in the lnCS of individual talar and cuneiform facets (Fig. 4 and Tables S4, 5). The talar facet/navicular ratio does not differ between *Pongo* and *H. sapiens* or *Gorilla*, whereas the cuneiform facets/navicular ratio discriminates all pairwise comparisons except *Pan* from *Gorilla* (Fig. 5 and Tables S5, 6). The tuberosity/navicular ratio differentiates all extant taxa. *Pongo* is characterised by an overall smaller tuberosity relative to the navicular size among the extant taxa, whereas *H. sapiens* has an intermediate ratio between *Pongo* and African great apes.

**Fig. 6:**
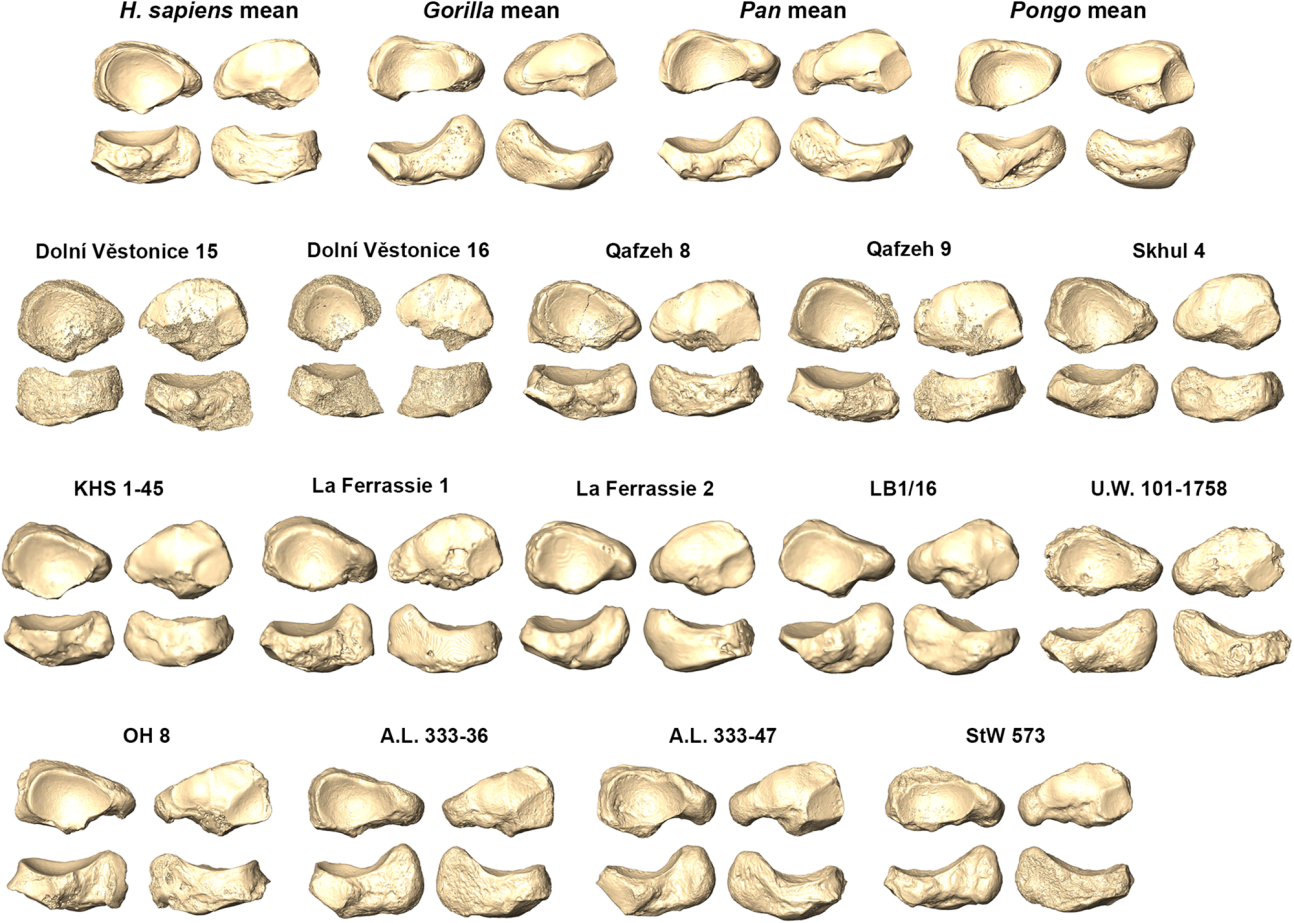
Navicular mean shape for each group. Remaining renderings illustrate fossil hominin naviculars in posterior (top left), anterior (top right), plantar (bottom left), and dorsal (bottom right) views.

Among fossil hominins, all *Homo* specimens (except for *H. naledi* and *H. floresiensis*) plot close to or within the *H. sapiens* range of variation (Fig. 3). OH 8 (variably attributed to *Homo habilis* or *Paranthropus boisei)* falls in the area of morphospace covered mainly by hunter-gatherers (Figs. 3, S3), and is assigned to *H. sapiens* with a 66.6% posterior probability (Table 2). OH 8 shows a tuberosity/navicular ratio approaching that of *H. sapiens* (Fig. 5 and Table S6), a reduced dorsoplantar length of the tuberosity compared to those of African great apes, a less concave talar facet, a less lateral-facing lateral cuneiform facet, and a relatively broader anteroposterior width of the navicular, although the latter is still quite tapered laterally as observed in great apes (Fig. 6). It is worth noting, however, that all the fossil hominins, except for the more recent Dolní Věstonice specimens, exhibit a more tapered lateral side compared to the combined living and archaeological *H. sapiens* sample. Australopiths, *H. naledi* and *H. floresiensis* are characterised by a more African great ape-like condition with a prominent tuberosity, a lateral-facing lateral cuneiform facet, and a less oval shaped and more concave talar facet relative to *H. sapiens* form (Fig. 6).

**Table 2:** Group affinity test based on Mahalanobis distance (D^2^).

	*H. sapiens* (%)	*Gorilla* (%)	*Pan* (%)	*Pongo* (%)
Dolní Věstonice 15	26.30	0.00	0.00	0.00
Dolní Věstonice 16	74.20	0.00	0.00	0.00
Qafzeh 8	9.90	0.00	0.00	0.00
Qafzeh 9	0.80	0.00	0.00	0.00
Skhul 4	32.50	0.00	0.00	0.00
Omo Kibish (KHS 1–45)	3.50	0.00	0.00	0.00
La Ferrassie 1	2.90	0.00	0.00	0.00
La Ferrassie 2	6.90	0.00	0.00	0.00
LB1/16	0.00	0.10	1.00	0.00
U.W. 101–1758	0.00	0.00	0.40	0.00
OH8	66.60	0.00	0.00	0.00
A.L. 333-36	0.00	0.10	0.00	0.00
A.L. 333-47	0.00	4.40	9.10	0.00
StW 573	0.00	2.20	16.30	0.00

(%) - Posterior probability of fossil specimens belonging to a genus based on the first three principal components scores of the Mahalanobis distance.

### Diversity in *Homo sapiens* and recent *Homo*, and OH8

**Fig. 7:**
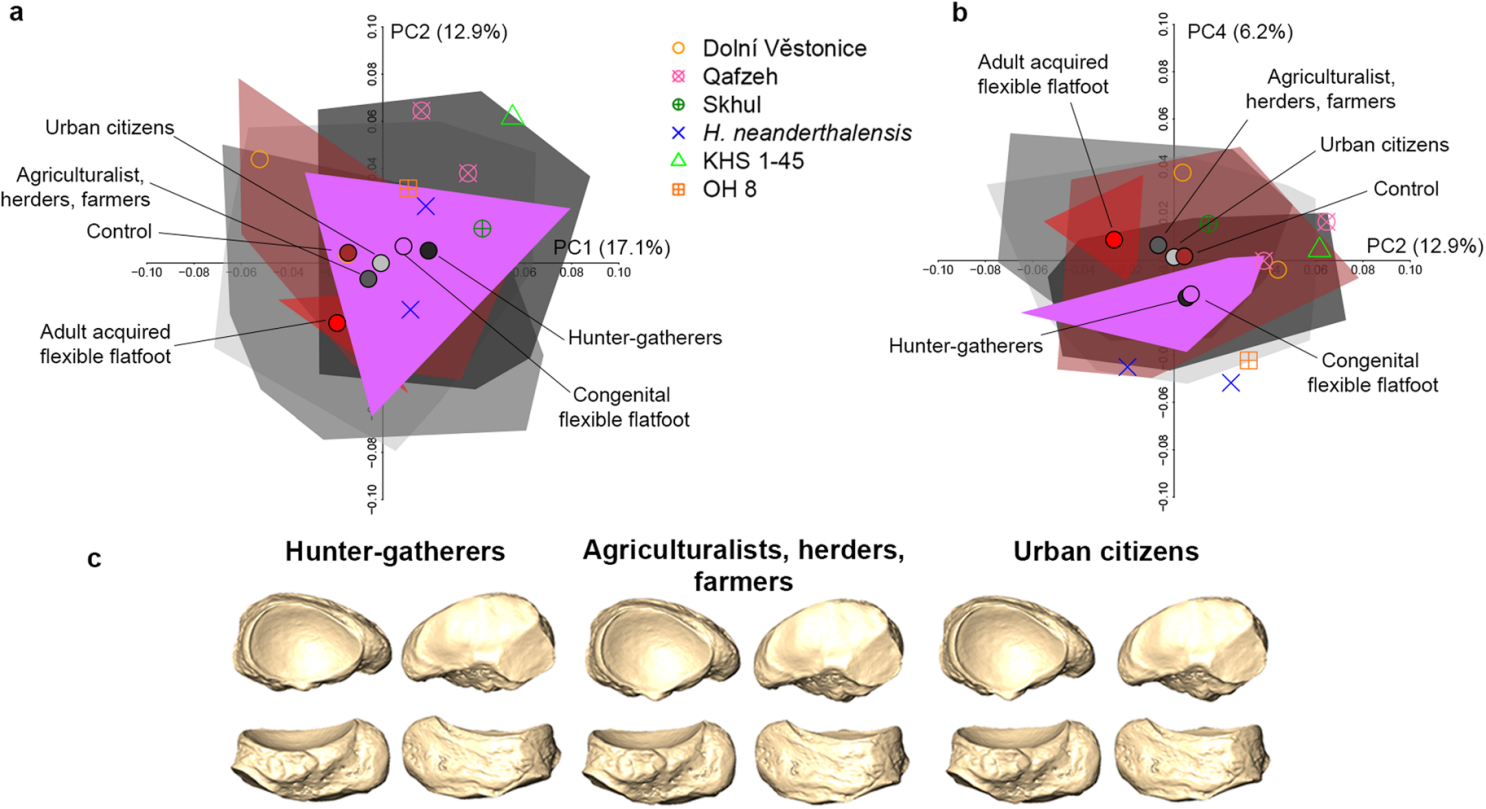
PCA plots of different *H. sapiens* groups, recent *Homo*, and OH8. PCA plots showing PC1 vs. PC2 (**a**) and PC2 vs. PC4 (**b**). Group means of living and archaeological *H. sapiens* are shown together with fossil *Homo* and OH 8 in plots. Mean navicular shape of hunter-gatherers, agriculturalists-herders-farmers, and urban citizens are represented in proximal (top left), distal (top right), plantar (bottom right), and dorsal (bottom left) views (**c**).

Subsistence strategy accounts for differences in the navicular shape of different *H. sapiens* groups (Fig. 7a, b and Table S7). Along PC1 (17.1%), hunter-gatherers differ from all other *H. sapiens* groups except from congenital flexible flatfoot (Table S7). These groups share a mediolaterally elongated talar facet, a navicular shortened in the dorsoplantar direction and a more lateral-facing lateral cuneiform facet (Fig. 7c). The adult acquired flexible flatfoot group significantly differs from the controls (PC2 and PC6), hunter-gatherers (PC1 to PC4), agriculturalists (PC3), post-industrials and congenital flexible flatfoot group (PC6; Table S7). Furthermore, congenital flexible flatfoot tends to separate from adult acquired flexible flatfoot along PC4 (Fig. 7b). Along PC1, late Upper Pleistocene fossil *H. sapiens* (Dolní Věstonice 15 and 16) segregate from late Middle Pleistocene/early Upper Pleistocene fossil *H. sapiens* (Qafzeh 8 and 9, Skhul 4, and Omo Kibish), where the latter show a relatively more mediolaterally elongated navicular with a reduced anteroposterior width (Fig. 6). Allometry does not account for these observed shape differences.

**Fig. 8:**
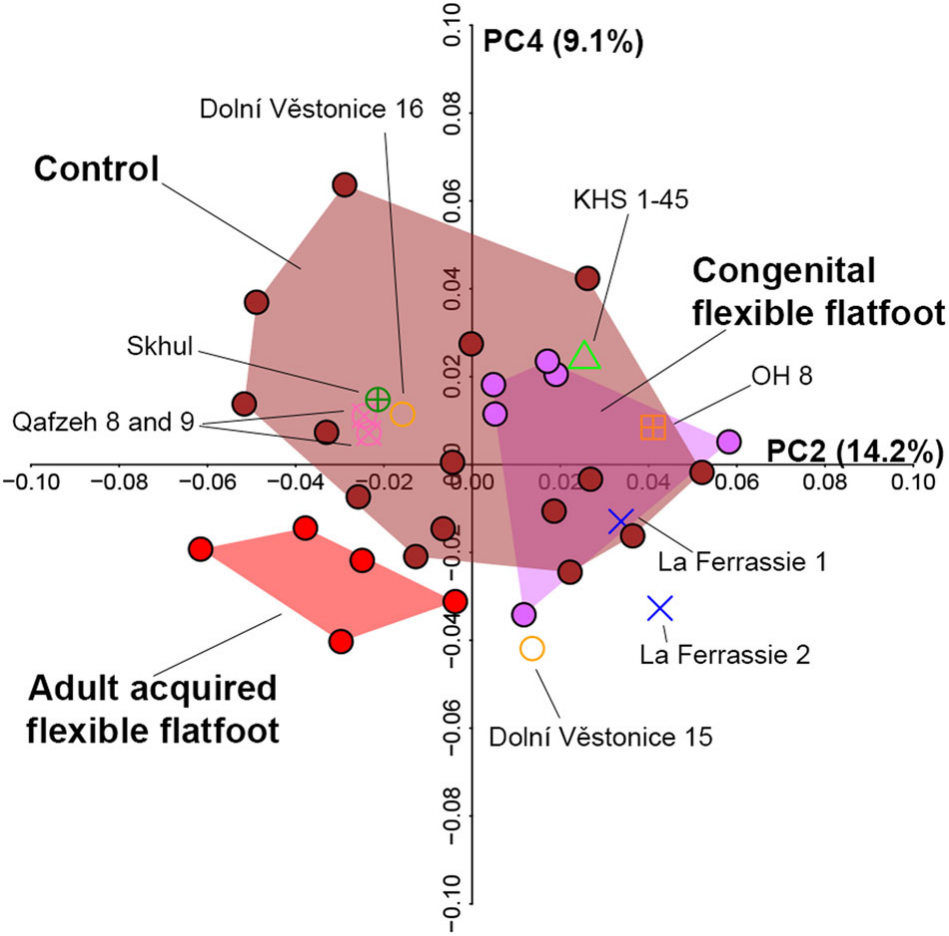
PCA plot of the living *H. sapiens* sample, recent *Homo*, and OH8. PCA plots showing PC2 vs. PC4. Fossil *H. sapiens, H. neanderthalensis* and OH 8 are projected in the shape space constructed from living *H. sapiens* (e.g., congenital flexible flatfoot, adult acquired flexible flatfoot, and control).

Figure 8 shows a shape-space PCA of the living *H. sapiens* sample, within which OH 8, *H. neanderthalensis* and the fossil *H. sapiens* have been projected. Most of the fossil specimens fall within the distribution of living *H. sapiens* that exhibit a clinically verified well-developed MLA (i.e., the control group). Interestingly, OH 8, the *H. neanderthalensis* specimen La Ferrassie 1 (but not La Ferrassie 2), Omo Kibish (KHS 1–45) and Dolní Věstonice 15 plot within or very close to the congenital flexible flatfoot group, but not close to the adult acquired flexible flatfoot group (Fig. 8).

## Discussion

The MLA is considered a hallmark of *H. sapiens* bipedalism. The navicular bone plays a pivotal role in arch formation and morphologically differs between clinically assessed normal arched feet and flatfooted feet, suggesting that bony morphology is involved in the structure of the MLA. Specifically, the navicular form in the adult acquired flexible flatfoot group differs from that in the control and congenital flexible flatfoot groups (Fig. 2a).

Adult acquired flexible flatfoot individuals are more narrowly distributed in the navicular morphospace than the control group, indicating that the spectrum of ‘normal’ variation exceeds the spectrum of variation that would fit into ‘abnormal’ as defined by the former (Figs. 2a, S1). It is possible that a larger sample of adult acquired flexible flatfoot individuals may expand the range of variation observed for this group in the present study, or that expanding the sample size of the control group would lead to greater overlap with the adult acquired flexible flatfoot group. In contrast, the asymptomatic congenital flexible flatfoot group falls entirely within the ‘normal’ spectrum of variation established by the control group in this study. This finding contributes to the clinical controversy regarding appropriate classification of congenital flexible flatfoot, suggesting that from an osteological perspective these individuals should not be identified as exhibiting pathological form, at least with respect to the navicular.

Congenital flexible flatfoot is a common condition, often inherited, and is generally pain-free. This condition is characterised by a normal arch when the foot is not loaded, and the results of this study corroborate the suggestion that it is arguably representative of a segment of variation in normal foot shape^20,37^. Instrumented gait analysis has shown conflicting results regarding the gait kinematics of children with flexible flatfoot^38,39^. For instance, children with flexible flatfoot (not specified if asymptomatic or not) exhibit a foot that behaves biomechanically similar to a standard arched foot during stance phase of gait^39^. Additionally, symptomatic flexible flatfoot shows abnormal foot function due to an altered pronation-supination dynamic of the hindfoot during stance phase based on ground reaction force measurements^40^. It has been reported that midfoot instability is present in adult acquired flexible flatfoot, and heel rise ability is impaired. Thus, the gait kinematics associated with adult acquired flexible flatfoot are compromised in transmitting loads from the rearfoot to the forefoot during the push-off period of stance phase^41^. Navicular shape may reflect these biomechanical observations in adult acquired flexible flatfoot by showing (1) a thickened plantar aspect suggesting an altered loading distribution likely directed more superiorly than inferiorly, potentially due to the flatness of the MLA; (2) more convexly curved cuneiform facets that could enable higher medial midfoot joint excursions in a dorsoplantar direction and as a by-product contribute to decreased bony stability of the midfoot; and (3) a more extensive tuberosity that may reflect bony changes related to the function of the *m. tibialis posterior*. Based on human electromyographic data acquired during walking, the tibialis posterior shows the largest bursts of activity shortly after heel contact and midstance events of the gait cycle, during which it acts to balance subtalar joint eversion moments in coordination with the peroneal muscles^42^. Thus, ‘hypertrophy’ of the tuberosity in the adult acquired flexible flatfoot group may be associated with the greater reliance on soft tissue structures (i.e., the tibialis posterior and associated ligaments attaching to the tuberosity) to sustain integrity of the arch. Furthermore, clinical data have shown that posterior tibial tendon dysfunction (e.g., elongation and degeneration of the posterior tibial tendon) is usually the cause of acquired flatfoot in adults^43^ and is associated with ligament collapse such as the spring ligament complex (i.e., superomedial and inferomedial calcaneonavicular ligaments)^44^. Further light cannot be shed on this issue since whether navicular shape variation is due to bone (re)modeling during life or whether it is a congenital predisposition is beyond the scope of our study. Additional studies are needed to determine whether bone shape is the primary cause, or whether it is a consequence of ligament and muscle-tendon alterations that also occur in adult acquired flexible flatfoot.

While expression of the MLA is reflected in navicular shape within living *H. sapiens*, a different interpretation emerges when the extant comparative sample of hominids is included. Navicular shape differences in hominids are partially driven by allometry, and appear to reflect locomotor behavioral, anatomical, and functional adaptations to a large extent since the phylogenetic signal is low. All *H. sapiens* groups are distinct from great apes primarily because of bipedalism, and indeed differences observed between adult acquired and congenital flexible flatfoot are swamped when comparing *H. sapiens* navicular form to that of the great apes (Fig. 3).

As far as the fossil hominin sample is concerned, the naviculars of *A. afarensis, A. prometheus, H. naledi*, and *H. floresiensis* plot outside the range of variation of *H. sapiens* naviculars and closer to distributions of those of African great apes, likely indicating a certain degree of arboreality in conjunction with terrestrial bipedalism. A more concave talar facet, as well as dorsally and convexly curved cuneiform facets in australopiths, *H. naledi*, and *H. floresiensis* (Fig. 6) could allow more midfoot mobility during push-off similar to what it is supposed to characterise the foot of great apes during propulsion^1,45,46^. A longer talar facet in a mediolateral direction associated with a lateral-facing lateral cuneiform facet in these fossil hominins (Fig. 6) could enhance mediolateral excursions in the medial midfoot and ultimately support a mobile hallux, likely reflecting a retention for pedal grasping during arboreal locomotion;^26^ this capability, at the same time, would be maladaptive in the presence of a human-like MLA.

While the arboreal interpretation of navicular morphology is consistent with the prediction of Holowka and Lieberman^47^ concerning anatomical foot adaptations in australopiths, it is partially surprising that two relatively recent *Homo* species (*H. floresiensis* and *H. naledi*) maintain some ape-like features in navicular shape. Several pedal features (e.g., curvature of the phalangeal shaft and declination of the talar head) present in *H. floresiensis* and *H. naledi* are shared with australopiths, suggesting these hominins may have retained selection on pedal grasping abilities and a weakly developed or absent MLA^22,23^. Australopiths and *H. naledi* also share with African great apes a prominent tuberosity with respect to overall navicular size (Fig. 5 and Table S6), which has been long interpreted as suggestive evidence against the presence of a MLA when the midfoot is involved in weight-bearing during midstance in terrestrial locomotion^24,26^. Alternatively, Prang^25^ suggested that a prominent tuberosity characterises more arboreal anthropoid taxa (e.g., Hylobates, and Ateles), likely indicating posterior tibialis development correlated with plantarflexion capability. However, based on a chimpanzee musculoskeletal model, O’Neill and colleagues^48^ suggested that a large physiological cross-sectional area for the tibialis posterior could be related to both plantarflexion and inversion that would be equally selectively important during arboreal modes. Since plantarflexion is achieved by many muscles, including the sizable triceps surae^49^, a prominent tuberosity may likely be signaling enhanced tibialis posterior recruitment to stabilise the foot against excessive eversion during early stance whether on the ground or a horizontal branch (or vertical trunk).

Aside from controversy on the taxonomic allocation of OH 8 (*H. habilis/P. boisei?*)^25,34–36^, our results (Fig. 3) are in line with studies suggesting that navicular shape of OH 8 resembles those of *H. sapiens*^24,25^ by showing a relatively smaller tuberosity, a less concave talar facet, a less lateral-facing lateral cuneiform facet, and a relatively anteroposteriorly broader navicular (Fig. 6). This is supported by the comparison with the talar head of OH 8, which is also more human-like than is the overall shape of the talus of OH 8^18^, suggesting functional adaptation of the medial column likely still favoring a configuration enabling an effective push-off during bipedal locomotion.

The navicular of OH 8 approaches the form of the naviculars of the hunter-gatherer *H. sapiens* group (Fig. 3). The hunter-gatherers differ from *H. sapiens* groups with a comparatively lower mobility level (e.g., urban, agriculturalist, herder, and farmer groups; Fig. 5 and Table S7) and, interestingly, plot near the congenital flexible flatfoot group. These two groups share a mediolaterally elongated talar facet, which may indicate broader mediolateral joint movements. Further, both groups are characterised by shorter naviculars in the dorsoplantar direction, particularly on the lateral side, and a more lateral-facing lateral cuneiform facet. These features, together with a more medially displaced talar head and neck^50^ suggest a more medial position of the navicular within the foot and more medial oriented medial and intermediate cuneiforms and, consequently, the same occurs also at the first two metatarsals and phalanges. Furthermore, a larger range of motion at the talonavicular joint may offer higher flexibility for adapting the midfoot to natural (not paved), uneven substrates (i.e., maximizing contact area between the plantar surface and the substrate) and likely a less distinct MLA when the foot is loaded as seen in congenital flexible flatfoot. Indeed, it has been demonstrated that most barefoot hunter-gatherers display wider and flatter foot strikes with respect to shod populations. This condition likely helps the foot conform to irregular natural surfaces (such as an unbroken forest floor) and promotes more tactile environmental stimuli, contributing to maintaining foot stability during substrate contacts^29,30,51^. Likely, foot joint kinematics and morphological foot features of unshod or minimally shod hunter-gatherers are more representative of the biomechanical circumstances in which the *H. sapiens* foot evolved compared to *H. sapiens* in modern industrialised societies, and in this sense, the similarity the former share with the congenital flexible flatfoot group is especially intriguing.

Altogether, results suggest that OH 8 and fossil *H. sapiens* (except for Dolní Věstonice 15) have a navicular configuration that may be associated with a segment of normal living *H. sapiens* variation in the expression of the MLA, comprising both individuals with a well-developed MLA and congenital flexible flatfoot (Fig. 7). No *Homo* fossils, including OH 8, fall in the morphospace occupied by the adult acquired flexible flatfoot. Regarding the fact that Dolní Věstonice 15 falls slightly outside the control and congenital flexible flatfoot ranges of variation, it is worth noting that this fossil is partially damaged (Fig. 6) and previous studies have even suggested that Dolní Věstonice 15 skeleton is pathological^52^.

Although *H. neanderthalensis* specimens plot outside the morphospace occupied by the adult acquired flexible flatfoot group (Fig. 8), both *H. neanderthalensis* specimens share with the adult acquired flexible flatfoot group a mix of characters such as a more posteriorly projecting tuberosity (especially visible in La Ferrassie 1), a more concave talar facet, and a greater anterior convexity of the navicular (i.e., the cuneiform facets; Fig. 6). This may suggest a different expression of the MLA in *H. neanderthalensis* with respect to those observed in the clinically diagnosed human groups analysed here. Recently, it has been suggested that the talus of *H. neanderthalensis* may reflect differences with *H. sapiens* likely related to a habitually pronated foot posture possibly due to higher body mass and/or higher mechanical stress^53^. Clinical studies on obese individuals have shown a prevalence of pronated foot posture, and also higher loading on the midfoot associated with greater degrees of flatfoot in these individuals^54^. In line with this, four out of five individuals from the adult acquired flexible flatfoot group in our sample are clinically overweight or obese. Although suggestive, a potentially different expression of the MLA in *H. neanderthalensis* attributable to their high body mass and/or bone plasticity related to particular biomechanical demands requires further validation by increasing the *H. neanderthalensis* sample size beyond that of the present study.

In conclusion, this study diachronically examined evolution of the MLA and variation as expressed by its keystone skeletal element, the navicular. The navicular bone reflects variation in the expression of the MLA in living *H. sapiens*, and *H. sapiens* mobility strategies and cultural adaptations (e.g., the use of shoes) may have influenced navicular shape. When considering extant hominids, navicular shape again appears strongly associated with locomotor behaviors. Thus, attention must be paid when inferring the expression of a MLA in fossil hominins based purely on comparisons between archaeological *H. sapiens* and great apes. This study underlines that the absence of the MLA in congenital flexible flatfoot (when the foot is loaded) does not imply pathological navicular shapes in these individuals, which overlap with those of the control group. In other words, the spectrum of normal variation in living *H. sapiens*, from the perspective of navicular shape, might include both feet with a well-developed MLA and also congenital flexible flatfoot. Nearly all the fossil *H. sapiens* sample falls within the spectrum of normal variation of the MLA, but not in the overlapping segment with congenital flexible flatfoot individuals, strongly suggesting the presence of a MLA in their feet. OH 8 and Omo Kibish, on the other hand, are in the overlapping segment between the control and congenital flexible flatfoot individuals, suggesting for these specimens the possible presence of a well-developed MLA or, alternatively, the absence of a MLA when the feet of these individuals were loaded during bipedal gait.

Finally, we hypothesise that the bones of the medial column of the foot (i.e., the talus, navicular, medial cuneiform, 1st metatarsal, and great toe) have shown great variability from past to present because they were not under strong selection for a high, i.e. well-developed, MLA. Importantly, our results suggest that a well-developed MLA may not be as vital as other mechanisms for stiffening the midfoot during the propulsion phase of bipedal gait^2,15,16,47^. However, it is important to note that this study focused only on a single bone of the medial column of the foot, and studies of other bones are needed to test further this hypothesis.

Ultimately, this study may help interpret foot shape in fossil hominins and *H. sapiens* from an archaeological context, which is particularly important when investigating evolution of the MLA and current variability.

## Methods

### Data collection

The sample consists of 357 extant and 14 extinct hominid naviculars (Tables S8, S9). The 3D surface renderings were acquired through laser or blue light scanning, computed tomography (CT) and micro-CT scanning. Regardless of the triangulation size defining surfaces, all digital techniques give comparable results and can be used in the same analysis^53,55^.

The extant sample includes 255 *H. sapiens*, 35 *Gorilla* (*G. beringei beringei*, *G. beringei graueri*, *G. gorilla*), 46 *Pan* (*P. troglodytes verus, Pan troglodytes troglodytes, P. troglodytes schweinfurthii*), and 21 *Pongo* (*P. abelii*, *P. pygmaeus*) (Tables S8, S9). African and Asian great apes were wild-caught specimens.

The samples (ten *Gorilla* and six *Pan*) from the Royal Museum for Central Africa, Tervuren, Belgium, were scanned in the UZ Leuven Department of Radiology (Leuven, Belgium) using a Siemens CT-SOM5 SPI medical scanner. Five *Gorilla* and six *Pan* from the Primate Collection of the Department of Comparative Anatomy of the National Museum of Natural History, Paris, France, were scanned in the Department of Radiology of the Pitié-Salpêtrière Hospital (Paris, France) using a Philips iCT256 medical scanner. Similar scanning parameters were used in both facilities: energy: 140 kVp; current: 120–253 µA; slice thickness: 0.67 mm; reconstruction increment: 0.3 mm. Raw data were reconstructed as 16-bit DICOM images using a bone reconstruction algorithm (i.e. a ‘sharp’ kernel)^56^. Three *P. t. schweinfurthii* specimens from —the primate collection of the University of Minnesota from the Gombe Chimpanzee Research Project, Minneapolis, MN, USA— were scanned at the Center for Clinical Imaging Research (CCIR), Department of Radiology at the University of Minnesota Medical School using a Siemens PETCT Biograph 64 medical scanner. This scanning facility uses the following parameters: energy: 140 kVp; current: 120 µA; slice thickness: 0.6 mm; reconstruction increment: 0.3 mm. The raw data were reconstructed as 16-bit 512 × 512 DICOM images using a bone reconstruction algorithm (i.e. an ‘H70 h’ convolution kernel). Five *Pongo* and four *Gorilla* were scanned with a micro-CT scanner Nikon XTH 225 ST HRCT laboratory scanning system at the University of Cambridge using the following parameters: energy = 125 kV; current = 135 μA; projections = 1080; filter = 0.1 mm copper; voxel resolution = 0.03–0.04 mm. Raw data were reconstructed as 16-bit tiff files. Non-human apes from the American Museum of Natural History (AMNH) and the Smithsonian Institution’s National Museum of Natural History (USNM) (Table S8) were scanned at the Molecular Imaging Center of the Department of Radiology in the Keck School of Medicine of the University of Southern California (Los Angeles, CA, USA) using a GE phoenix nanotom m system with the following parameters: energy = 100 or 120 kV; current = 80 or 190 μA; projections = 1440 or 1800/360°; filter = 0.1 mm copper; voxel resolution = 0.03–0.07 mm. Some of the extant ape surface models can be found at www.MorphoSource.org.

The living *H. sapiens* sample (above the age of 18 years) was selected after a clinical assessment of the foot type (Table S9). The control and congenital flexible flatfoot samples were acquired via Cone beam weight-bearing CT (WBCT) scans (CurveBeam, Philadelphia, USA) at the Department of Orthopedics, School of Medicine, University of Colorado, USA. All scans were obtained originally for standard clinical care purposes and then retrospectively screened and collected for use in this study with approval by the Colorado Multiple Institutional Review Board and all ethical regulations relevant to human research participants were followed. The control scans were from patients who underwent WBCT scans for reasons other than trauma and deformities in the foot and ankle. All patients in this group had a well-developed LA arch under weight-bearing with a normal hindfoot alignment. Congenital flexible flatfoot was defined when a patient was born with bilateral flatfoot and had both a flat arch, and a flexible valgus hindfoot when the WBCT scans were taken. Clinico-radiological assessment and WBCT scans (‘OnSight 3D Extremity System’, Carestream, Rochester, NY) of five adult acquired flexible flatfoot were performed at Istituto Ortopedico Rizzoli - IOR, Italy. These WBCT scans were taken in accordance with relevant Italian National guidelines and regulations, and informed consent was obtained from all patients. These five patients were diagnosed as having severe flat feet with limited foot function, and four were overweight or obese (BMI females: 27.9, 20.5, 26.9, 34.4; BMI male: 32).

The archaeological *H. sapiens* sample includes adult individuals spanning the last 12 Ka and are characterised by different mobility and subsistence strategies (Table S9), including urban citizens, agriculturalists, herders, farmers and hunter-gatherers. Surfaces of the majority of these naviculars were acquired with an ARTEC Space Spider 3D structured light laser scanner (0.1 mm resolution) at their host institutions or at the Department of Cultural Heritage, University of Bologna (Italy), except for adult individuals from South Africa that are housed in the Raymond A. Dart Collection of Human Skeletons at the University of the Witwatersrand, Johannesburg, South Africa. Naviculars from the Dart collection were CT scanned at the Charlotte Maxeke Johannesburg Academic Hospital (South Africa) using a Philips Brilliance 16 P medical CT scanner. The U.H.R. 0.5 (Philips Healthcare, Andover, MA, USA) protocol used for scanning had the following parameter settings: energy: 140 kVp; current: 253 mA; slice thickness: 0.67 mm; reconstruction increment: 0.3 mm. Raw data were reconstructed as 16-bit 512 × 512 DICOM images using a bone reconstruction algorithm (i.e., a ‘sharp’ c kernel). Moreover, the specimens housed at Florisbad Quaternary Research Station of the National Museum of Bloemfontein, South Africa, were scanned at the Microfocus X-ray Computed Tomography Facility in the Evolutionary Studies Institute of the University of the Witwatersrand (Johannesburg, South Africa) using a Nikon Metrology XTH 225/320 LC dual source industrial μCT system. The scanning protocol included the following parameter settings: energy: 90 kVp; current: 220 µA; projections: 1000; filter: 0.5 mm copper; voxel dimension: 0.026–0.049 mm. Raw data were reconstructed as 16-bit DICOM images using Nikon image reconstruction software 3D Pro^56^.

Fossil naviculars (Table S10) come from different sources. Dolní Vĕstonice 15 and 16, Qafzeh 8 and 9, and Skhul 4 were scanned with a BIR ACTIS high-resolution CT scanner (130 kV, 100 μA, isometric voxels of 0.029–0.054 mm) at the Department of Human Evolution, Max Planck Institute for Evolutionary Anthropology (Leipzig, Germany). Omo-Kibish 1, A.L. 333-36, A.L. 333-47, U.W. 101–1758, and OH 8 (cast) were scanned with a Creaform Go!SCAN 20 with a resolution of 0.1 mm; a high-resolution cast of the LB1/16 navicular was scanned with a Next Engine desktop laser scanner; high resolution scanning of the StW 573 navicular was conducted with an Artec Space Spider, a blue light scanner; CT-scans of La Ferrassie 1 and 2 (voxels of 0.21–0.25 mm) were acquired from the National History Museum Paris, Anthropology Collection.

Digital 3D models of naviculars virtually acquired with CT or micro-CT scans were generated in Avizo v. 9.2 (Thermo Fisher Scientific, Waltham) through isosurface reconstructions. The overall sample consists of left naviculars or mirrored right naviculars (e.g., if the left navicular was missing or damaged). Only one navicular per individual was used for geometric morphometric analyses.

### Statistics and reproducibility

A 3D template of 85 landmarks and semi-landmarks (Fig. 1; Table S11) was applied to the digital 3D models (targets) in Viewbox v. 4 (dHAL software, Kifissia) allowing semi-landmarks to slide along the curves and surfaces to minimize thin-plate spline (TPS) bending energy between the target and template, lastly obtaining geometrically homologous semi-landmarks of curves and surfaces^57^. Digital reconstruction based on TPS interpolation of the missing semi-landmarks of fragmented fossil specimens was performed in Viewbox v. 4 following the procedure described in Sorrentino and colleagues^18,58^. In particular, missing portions of the naviculars of Dolní Vĕstonice 15 and 16, and that of Qafzeh 9, were reconstructed based on TPS interpolations with the living and archaeological *H. sapiens* mean. In contrast, the reconstruction of StW 573 was based on the TPS interpolation with the extant sample mean after testing whether using different means (i.e., the *Pan* mean, *Gorilla* mean, *Pongo* mean, or living and archaeological *H. sapiens* mean) would have affected the final result.

Cartesian coordinates (Supplementary Data 1) were imported in R v. 4.0.5^59^ and four separate GPAs (R package geomorph v. 4.0.0^60^) were performed to scale, rotate and translate the coordinates. At the same time, semi-landmarks were allowed to slide again with each recursive update of the Procrustes consensus of each subset within the overall sample^61^. The first GPA was conducted on the living *H. sapiens* sample comprising congenital flexible flatfoot, adult acquired flexible flatfoot, and the control groups. The second GPA was performed on the overall sample (great apes, *H. sapiens*, and fossil hominins) and used to inspect outliers by using the R package geomorph v. 4.0.0^60^. This GPA was repeated after removing nine outliers (i.e., one male individual from Bologna, two male individuals from Sotho, one individual of  unknown sex from Roccapelago, one male and one female individual from Via Orfeo, three individuals of unknown sex from Al Khiday) from the archaeological *H. sapiens* sample (Table S9). The third GPA was performed on the living and archaeological *H. sapiens* sample and navicular fossils of Dolní Vĕstonice 15 and 16, Qafzeh 8 and 9, Skhul 4, OH 8, Omo-Kibish 1, and La Ferrassie 1 and 2. The latter list of fossils was used for the fourth GPA, which included only the living *H. sapiens*. Procrustes coordinates were used to explore shape variation through PCA, and when included, fossils were projected into the principal coordinate space of the extant sample. Accordingly, PC scores of fossils were predicted by multiplying the matrix of shape variables of the fossils with the covariance matrix of the extant sample using R-code provided in^18,58,62–64^.

Several analyses were performed to investigate specific findings of the present work and adhere to assumptions (e.g., sample size, normal distribution) of specific tests.

Size was evaluated as the natural logarithm of centroid size (lnCS). Centroid size (CS) is calculated as the square root of the summed squared distances between each landmark and semi-landmark and the centroid of (semi)landmarks configuration. Several CSs were calculated for the overall navicular and each navicular feature (i.e., the talar facet, cuneiform facets, and tuberosity, respectively; Supplementary Data 1). Further, a ratio between each navicular feature and overall navicular lnCS was calculated. For instance, individuals with a relatively larger navicular tuberosity should have larger ratio values. Differences among groups in lnCS and ratios were inspected via Kruskal-Wallis tests with Bonferroni corrections, and Mann–Whitney U intergroup comparisons, when data did not pass a normality test. Otherwise, one-way ANOVA with the Tukey post hoc test were used to determine the statistical significance of group comparisons. Size differences were lastly visualised using boxplots^62^ (see Figs. 2, 4, 5).

Size and shape association, i.e., allometry, along the PCs was evaluated through Pearson product-moment correlation coefficients (r) of the scores of the PCs (shape variables) and navicular lnCS. When a high correlation coefficient (r) was obtained for the shape variables with lnCS in the analysis with great apes, *H. sapiens*, and fossil hominins, a partial least squares (PLS) regression was performed between Procrustes coordinates and lnCS to measure degrees of covariation.

We used Procrustes ANOVA to test the null hypothesis that genus, lnCS, and genus:lnCS interaction terms did not explain variation in navicular shape using the R package geomorph v. 4.0.0^60^. Whereas shape differences among *H. sapiens* archaeological and living groups were tested via a permutation (1.000) of the distance between group means using the R package Morpho v. 2.8^65^.

The role of phylogeny in navicular shape variation among extant taxa was evaluated using the multivariate version of the Blomberg’s K Statistic (Kmult^66^) based on the Procrustes mean of each species and subspecies of the extant taxa (9 tips: *G. beringei beringei*, *G. beringei graueri*, *G. gorilla*, *P. troglodytes verus*, *Pan troglodytes troglodytes*, *P. troglodytes schweinfurthii*, *P. abelii*, *P. pygmaeus*, *H. sapiens*) using the function ‘physignal’ in the R package geomorph v. 4.0.0^60^. The Kmult can detect phylogenetic signals in high-dimensional multivariate traits like shapes whose dimensionality exceeds the number of tips in the tree (i.e., number of species)^66^, although the authors recognize the limitations of implementing this method with only 9 species. The consensus tree used in this function was freely downloaded from the 10kTrees website following the link https://10ktrees.nunn-lab.org/. Interpretation is based on the Kmult<1, indicating that the phylogenetic signal is less than expected under a Brownian motion model of evolution^67^.

Affinities of fossil hominins with extant taxa were calculated by computing the posterior probability that each fossil hominin belongs to any extant taxon based on the Mahalanobis distance (D^2^)^68,69^, using the ‘typprobClass’ function in the R package Morpho v. 2.8^65^.

All statistical tests were performed in R v. 4.0.5^59^.

### Reporting summary

Further information on research design is available in the Nature Portfolio Reporting Summary linked to this article.

### Supplementary information

Supplementary Information

Description of Additional Supplementary Files

Supplementary Data 1

Reporting Summary

## Data Availability

All data needed to evaluate the conclusions in the paper are present in the manuscript and/or the Supplementary Information. Digital models of non-human apes from the AMNH and USNM are available from www.MorphoSource.org (Anthropoid Primate Feet). Other 3D models of naviculars are available from the authors; however, restrictions apply to the availability of these data since they were used explicitly under license for the current study and are not publicly accessible. The 3D geometric morphometric data (i.e., landmarks coordinates) are available in the Supplementary Data 1 File.
