# Supplementary material for: Morphological and evolutionary insights into the keystone element of the human foot’s medial longitudinal arch
Source: Commun Biol. 2023 Oct 19;6:1061. doi: 10.1038/s42003-023-05431-8 (PMC10587292; doi:10.1038/s42003-023-05431-8)
Supplement: Supplementary file 1 — Supplementary Information [file 42003_2023_5431_MOESM1_ESM.pdf]

Supplementary Information

Morphological and evolutionary insights into the keystone element of the human foot's medial longitudinal arch

Rita Sorrentino[1], Kristian J. Carlson[2,3], Caley M. Orr[4,5], Annalisa Pietrobelli[1], Carla Figus[6], Shuyuan Li[7], Michele Conconi[8], Nicola Sancisi[8], Claudio Belvedere[9], Mingjie Zhu[7], Luca Fiorenza[10], Jean-Jacques Hublin[11,12], Tea Jashashvili[2,13], Mario Novak[14], Biren A. Patel[2,15], Thomas C. Prang[16], Scott A. Williams[3,17,18], Jaap P. P. Saers[19], Jay T. Stock[20], Timothy Ryan[21], Mark Myerson[7], Alberto Leardini[9], Jeremy DeSilva[22], Damiano Marchi[23,18,*], Maria Giovanna Belcastro[1,*], Stefano Benazzi[6,*]

[1]*Department of Biological, Geological and Environmental Sciences, University of Bologna, Bologna 40126, Italy*
[2]*Department of Integrative Anatomical Sciences, Keck School of Medicine, University of Southern California, Los Angeles 90033, USA*
[3]*Evolutionary Studies Institute, University of the Witwatersrand, Johannesburg, WITS 2050, South Africa*
[4]*Department of Cell and Developmental Biology, University of Colorado School of Medicine, Aurora, CO 80045, USA*
[5]*Department of Anthropology, University of Colorado Denver, Denver CO 80217, USA*
[6]*Department of Cultural Heritage, University of Bologna, Ravenna 48121, Italy*
[7] *Department of Orthopaedic Surgery, University of Colorado, Denver, CO, USA*
[8]*Department of Industrial Engineering, Health Sciences and Technologies, Interdepartmental Centre for Industrial Research (HST–ICIR), University of Bologna, Bologna 40136, Italy*
[9]*Laboratory of Movement Analysis and Functional Evaluation of Prostheses, IRCCS Istituto Ortopedico Rizzoli, Bologna, Italy*
[10]*Monash Biomedicine Discovery Institute, Department of Anatomy and Developmental Biology, Monash University, Melbourne, VIC 3800, Australia*
[11]*Chaire Internationale de Paléoanthropologie, CIRB (UMR 7241–U1050), Collège de France, Paris, France*
[12] Max Planck Institute for Evolutionary Anthropology, Leipzig 04103, Germany
[13]*Department of Geology and Paleontology, Georgian National Museum, Tbilisi 0105, Georgia*
[14]*Centre for Applied Bioanthropology, Institute for Anthropological Research, Zagreb 10000, Croatia*
[15]*Human and Evolutionary Biology Section, Department of Biological Sciences, University of Southern California, Los Angeles 90089, USA*
[16]*Department of Anthropology, Washington University in St. Louis, St. Louis, MO 63130, USA*
[17]*Center for the Study of Human Origins, Department of Anthropology, New York University, New York 10003, USA*
[18]*Centre for the Exploration of the Deep Human Journey, University of the Witwatersrand, Johannesburg, Wits 2050, South Africa*
[19]*Naturalis Biodiversity Center, 2333 CR Leiden, the Netherlands*
[20]*Department of Anthropology, Western University, London, Ontario N6A 3K7, Canada*
[21]*Department of Anthropology, The Pennsylvania State University, State College, PA 16802, USA*
[22]*Department of Anthropology, Dartmouth College, Hanover, NH* 03755, *USA*
[23]*Department of Biology, University of Pisa, Pisa 56126, Italy*

*equal contribution.

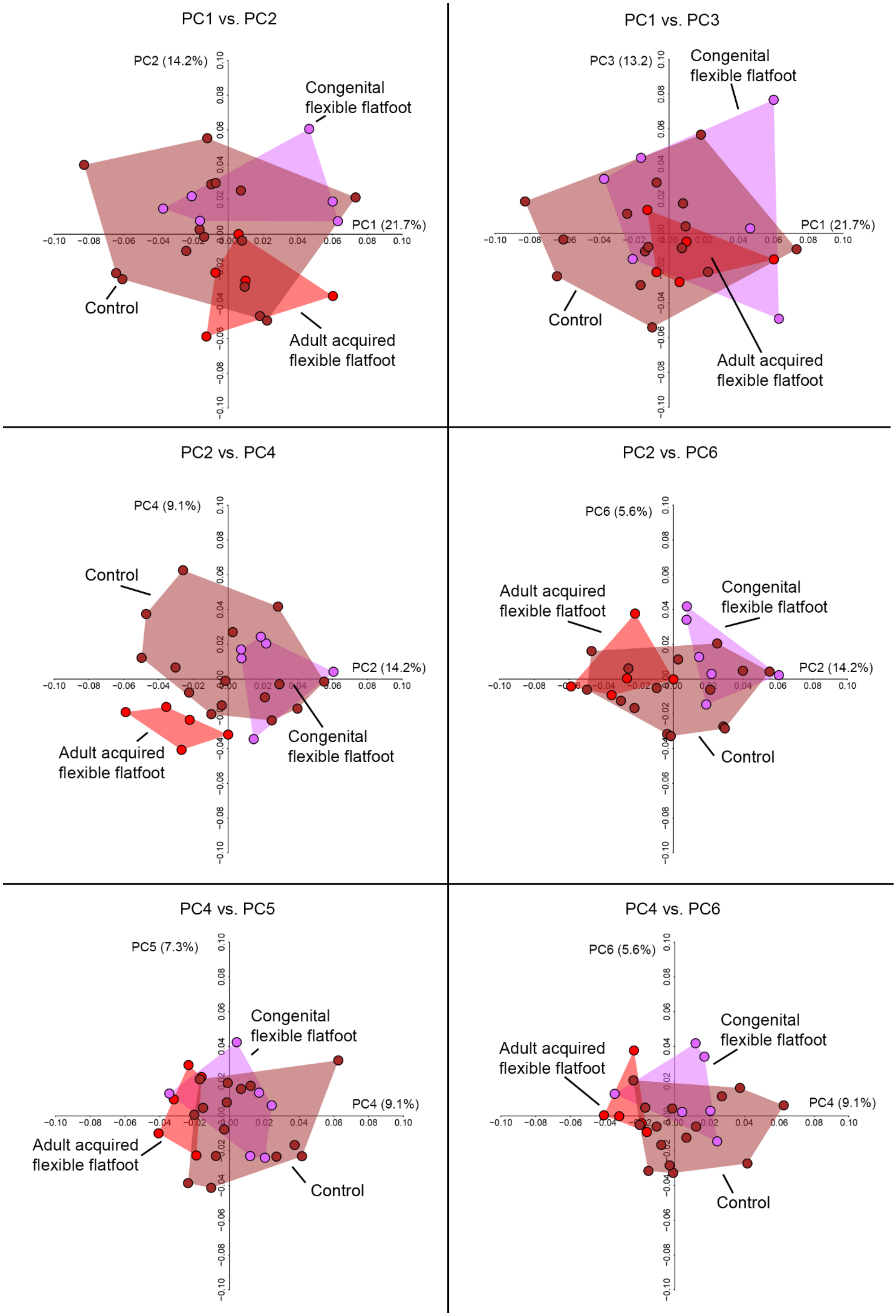

Fig. S1. Principal component analysis (PCA). PCA plots showing selected combinations of the first 6 PCs.

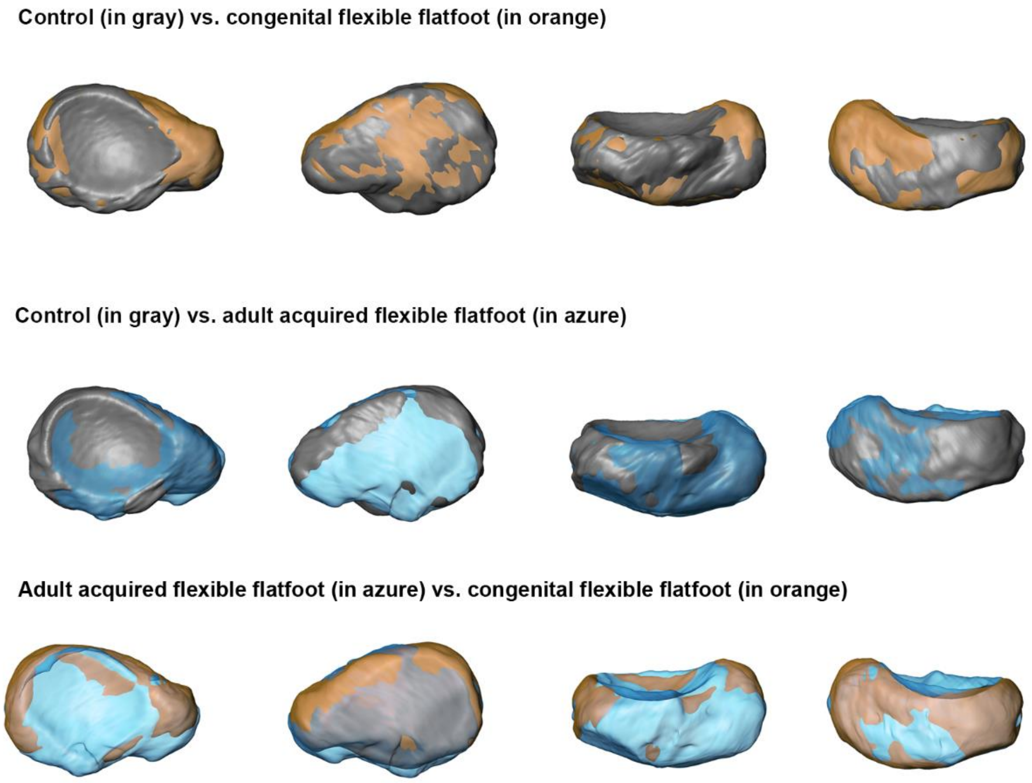

Fig. S2. The 3D surface superimposition of means between all possible pairs (i.e., control and congenital flexible flatfoot at the top; control and adult acquired flexible flatfoot at the middle; and congenital flexible flatfoot and adult acquired flexible flatfoot at the bottom). Shapes of naviculars are shown in proximal, distal, plantar, and dorsal views from left to right.

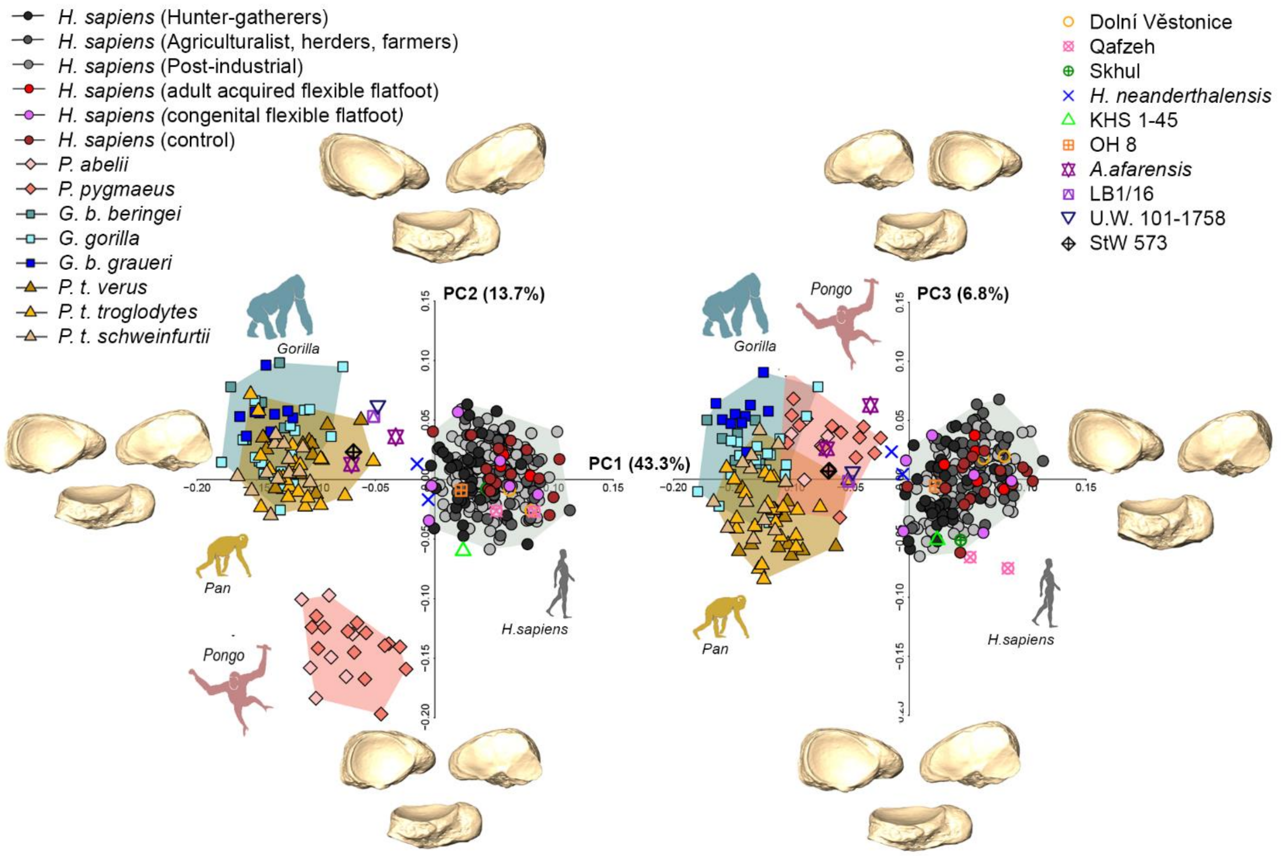

Fig. S3. Principal component analysis (PCA). The PCA plot shows PC1 vs. PC2 (left) and PC1 vs. PC3 (right) variance distributions for navicular shapes of great apes (i.e., *Pan*, *Gorilla,* and *Pongo*) and the varied *H. sapiens* groups (i.e., different subsistence activities - hunter-gatherers, agriculturalists, and post-industrial - and clinical classifications - congenital flexible flatfoot, adult-acquired flexible flatfoot, and normal or control). 3D surface renderings representing the extreme negatively and positively loaded shapes of variance along PC 1, 2, 3, and 4 are provided in proximal (top left), distal (top right), and plantar (bottom) views.

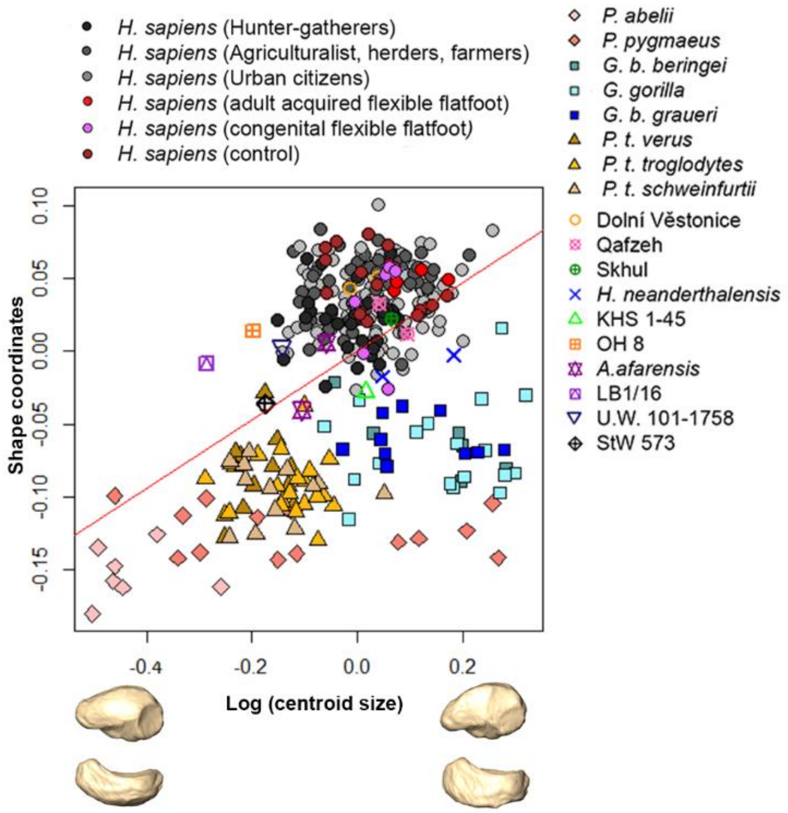

Fig. S4 – Partial Least squares regression (r-PLS = 0.410, p = 0.001) between lnCS and shape coordinates.

Table S1. Statistical significance of the principal components (PCs 1-6) of navicular shape variance using Permutation tests among living *H. sapiens* groups clinically classified as congenital flexible flatfoot, adult-acquired flexible flatfoot, and control (no flatfoot diagnosis).

	PC1		PC2		PC3		PC4		PC5		PC6	
	CTRL	CFF	CTRL	CFF	CTRL	CFF	CTRL	CFF	CTRL	CFF	CTRL	CFF
CFF	0.171		0.165		0.236		0.920		0.463		0.039*	
AAFF	0.304	0.846	0.064	0.005*	0.546	0.139	0.016*	0.029*	0.412	0.952	0.251	0.511

*Significant *p*-value ($p < 0.05$).
Abbreviations: CFF, congenital flexible flatfoot; AAFF, adult acquired flexible flatfoot; CTRL, control.

Table S2. Means and standard deviation (SD) of lnCS, relative lnCS of different anatomical parts, and the ratios of lnCS of the different anatomical parts and overall navicular of living *H. sapiens* groups.

	**Control** (UCSM)		**Congenital flexible flatfoot** (UCSM)		**Adult acquired flexible flatfoot** (IOR)	
Feet n°	15		6		5	
**lnCS**	Mean	SD	Mean	SD	Mean	SD
Overall navicular	4.86	0.08	4.86	0.03	4.92	0.05
Talar facet	4.04	0.08	4.07	0.05	4.11	0.05
Cuneiform facets	4.31	0.08	4.31	0.03	4.37	0.04
Tuberosity	3.22	0.08	3.24	0.08	3.32	0.08
**Ratio %**						
Talar facet/ navicular	83.21	0.78	83.80	0.93	83.47	0.53
Cuneiform facets/ navicular	88.68	0.58	88.71	0.45	88.88	0.42
Tuberosity/ navicular	66.21	0.87	66.25	1.31	67.55	1.02

UCSM = University of Colorado School of Medicine, USA; IOR = Istituto Ortopedico Rizzoli, Italy.

Table S3. Statistical significance of the principal components (PCs 1-3) of navicular shape variance using Permutation tests among great apes (i.e., *Pan*, *Gorilla*, and *Pongo*) and *H. sapiens*. The *H. sapiens* group includes living and archaeological *H. sapiens* samples in this study (i.e., different subsistence activities - hunter-gatherers, agriculturalists, post-industrial - and clinical classifications - congenital flexible flatfoot, adult acquired flexible flatfoot, and normal or control).

	PC1			PC2			PC3		
	Homo sapiens	*Pan*	*Gorilla*	*Homo sapiens*	*Pan*	*Gorilla*	*Homo sapiens*	*Pan*	*Gorilla*
Pongo	0.001*	0.028*	0.001*	0.001*	0.001*	0.001*	0.001*	0.001*	0.802
Homo sapiens		0.001*	0.001*		0.335	0.001*		0.001*	0.001*
Pan			0.371			0.006*			0.001*

*Significant *p*-value ($p < 0.05$).

Table S4. Means and standard deviation (SD) of lnCS of the different anatomical parts and overall navicular for great apes, *Homo sapiens* (i.e., combined living and archaeological *H. sapiens*), and fossil hominins.

	Overall navicular		Talar facet		Cuneiform facets		Tuberosity	
	Mean	SD	Mean	SD	Mean	SD	Mean	SD
Pongo	4.61	0.26	3.87	0.25	4.1	0.25	2.73	0.30
Pan	4.67	0.07	3.88	0.08	4.1	0.08	3.14	0.08
Gorilla	4.96	0.12	4.19	0.12	4.35	0.12	3.45	0.13
Homo sapiens	4.85	0.08	4.06	0.09	4.29	0.08	3.18	0.11
Dolní Věstonice 15	4.81	-	4.00	-	4.28	-	3.16	-
Dolní Věstonice 16	4.86	-	4.03	-	4.31	-	3.21	-
Qafzeh 8	4.91	-	4.17	-	4.42	-	3.19	-
Qafzeh 9	4.86	-	4.11	-	4.37	-	3.13	-
Skhul 4	4.88	-	4.10	-	4.39	-	3.17	-
Omo Kibish (KHS 1-45)	4.84	-	4.09	-	4.33	-	3.01	-
La Ferrassie 1	5	-	4.19	-	4.43	-	3.5	-
La Ferrassie 2	4.87	-	4.08	-	4.35	-	3.18	-
LB1/16	4.53	-	3.76	-	3.95	-	2.97	-
U.W. 101-1758	4.68	-	3.89	-	4.08	-	3.17	-
OH8	4.62	-	3.83	-	4.05	-	2.96	-
A.L. 333-36	4.76	-	3.96	-	4.10	-	3.34	-
A.L. 333-47	4.71	-	3.92	-	4.11	-	3.23	-
StW 573	4.65	-	3.85	-	4.04	-	3.15	-

Table S5. Kruskal-Wallis or ANOVA tests (in parenthesis) and subsequent pairwise tests (Mann-Whitney U or Tukey post hoc) for differences in lnCS and ratios of the different anatomical parts and overall navicular for great apes and *Homo sapiens* (i.e., combined living and archaeological *H. sapiens*).

	H. sapiens *vs. Gorilla*	*H. sapiens* *vs. Pan*	*H. sapiens* *vs. Pongo*	*Gorilla* *vs. Pan*	*Gorilla vs.* *Pongo*	*Pan vs.* *Pongo*
**Size**						
Overall navicular (F = 92.59, Df = 3, p < 0.001)	<0.001*	<0.001*	<0.001*	<0.001*	<0.001*	0.171
Talar facet ($\chi2$ = 121.24, Df = 3, p < 0.001)	<0.001*	<0.001*	<0.001*	<0.001*	<0.001*	0.198
Cuneiform facets ($\chi2$ = 112.17, Df = 3, p < 0.001)	0.006*	<0.001*	<0.001*	<0.001*	<0.001*	0.364
Tuberosity ($\chi2$ = 107.31, Df = 3, p < 0.001)	<0.001*	0.001*	<0.001*	<0.001*	<0.001*	<0.001*
**Ratio**						
Navicular/ talar facet (F = 17.9, Df = 3, p < 0.001)	<0.001*	<0.001*	0.578	<0.001*	0.154	<0.001*
Navicular/ cuneiform facet ($\chi2$ = 88.22, Df = 3, p < 0.001)	<0.001*	0.019*	0.020*	0.824	<0.001*	<0.001*
Navicular/ tuberosity ($\chi2$ = 153.09, Df = 3, p < 0.001)	<0.001*	<0.001*	<0.001*	<0.001*	<0.001*	<0.001*

*Significant *p*-value (*p* = < 0.05).

Table S6. Means and standard deviations (SD) of the ratios of lnCS of the different anatomical parts and overall navicular for great apes, *Homo sapiens* (i.e., combined living and archaeological *H. sapiens*), and fossil hominins.

	Talar facet/ navicular		Cuneiform facets/ navicular		Tuberosity/ navicular	
	Mean	SD	Mean	SD	Mean	SD
Pongo	84.00	0.93	88.9	0.77	59.08	3.26
Pan	83.23	0.69	87.78	0.61	67.23	1.12
Gorilla	84.43	0.61	87.72	0.58	69.52	1.46
Homo sapiens	83.79	0.76	88.51	0.53	65.64	1.54
Dolní Věstonice 15	83.22	-	89.09	-	65.85	-
Dolní Věstonice 16	82.96	-	88.67	-	65.99	-
Qafzeh 8	84.89	-	89.94	-	64.99	-
Qafzeh 9	84.53	-	89.95	-	64.29	-
Skhul 4	84.03	-	89.92	-	64.97	-
Omo Kibish (KHS 1-45)	84.59	-	89.54	-	62.31	-
La Ferrassie 1	83.79	-	88.57	-	69.91	-
La Ferrassie 2	83.76	-	89.35	-	65.26	-
LB1/16	82.92		87.11		65.46	-
U.W. 101-1758	83.10	-	87.28	-	67.79	-
OH8	82.85	-	87.74	-	64.09	-
A.L. 333-36	83.28	-	86.22	-	70.07	-
A.L. 333-47	83.23	-	87.25	-	68.56	-
StW 573	82.97	-	87.00	-	67.79	-

Table S7. Statistical significance of the principal components (PCs 1-6) of navicular shape variance using Permutation tests among different living and archaeological *H. sapiens* groups (i.e., different subsistence activities - hunter-gatherers, agriculturalists, post-industrial - and clinical classifications - congenital flexible flatfoot, adult acquired flexible flatfoot, and normal or control).

		Control	Congenital flexible fatfoot	Hunter-gatherers	Agriculturalist, herders, farmers	Adult acquired flexible flatfoot
**PC1**	Congenital flexible fatfoot	0.167				
	Hunter-gatherers	0.001*	0.483			
	Agriculturalist, herders, farmers	0.351	0.336	0.001*		
	Adult acquired flexible flatfoot	0.781	0.169	0.015*	0.394	
	Post-industrial	0.109	0.529	0.003*	0.329	0.201
**PC2**	Congenital flexible fatfoot	0.853				
	Hunter-gatherers	0.928	0.901			
	Agriculturalist, herders, farmers	0.182	0.268	0.064		
	Adult acquired flexible flatfoot	0.038*	0.056	0.023*	0.162	
	Post-industrial	0.609	0.571	0.372	0.148	0.051
**PC3**	Congenital flexible fatfoot	0.298				
	Hunter-gatherers	0.031*	0.655			
	Agriculturalist, herders, farmers	0.020*	0.714	0.940		
	Adult acquired flexible flatfoot	0.466	0.142	0.029*	0.028*	
	Post-industrial	0.136	0.837	0.162	0.115	0.080
**PC4**	Congenital flexible fatfoot	0.100				
	Hunter-gatherers	0.009*	0.881			
	Agriculturalist, herders, farmers	0.444	0.026*	0.001*		
	Adult acquired flexible flatfoot	0.517	0.081	0.023*	0.811	
	Post-industrial	0.951	0.080	0.001*	0.123	0.421
**PC5**	Congenital flexible fatfoot	0.047*				
	Hunter-gatherers	0.965	0.026*			
	Agriculturalist, herders, farmers	0.016*	0.540	0.001*		
	Adult acquired flexible flatfoot	0.165	0.676	0.134	0.990	
	Post-industrial	0.002*	0.628	0.001*	0.695	0.892
**PC6**	Congenital flexible fatfoot	0.777				
	Hunter-gatherers	0.003*	0.007*			
	Agriculturalist, herders, farmers	0.023*	0.056	0.171		
	Adult acquired flexible flatfoot	0.032*	0.042*	0.775	0.340	
	Post-industrial	0.005*	0.027*	0.458	0.371	0.516

*Significant *p*-value ($p = < 0.05$).

Table S8. Extant comparative sample.

Species	Sex			Total	Institution/Collection
	Females	Males	Unknown		
Homo sapiens	89	105	61	255	see Supplementary Table 5
Pan troglodytes verus	5	6	-	11	AMNH, USNM
Pan troglodytes troglodytes	9	10	3	22	AMNH, USNM
Pan troglodytes schweinfurthii	3	5	5	13	AMNH, USNM, NMNHP, Gombe, Tervuren
Gorilla beringei beringei	2	4	-	6	AMNH, USNM
Gorilla beringei graueri	6	3	1	10	NMNHP, Tervuren
Gorilla gorilla	7	5	7	19	AMNH, USNM, DAUC, NMNHP, Gombe, Tervuren
Pongo abelii	6	1	-	7	USNM
Pongo pygmaeus	5	4	5	14	USNM, DAUC

AMNH - American Museum of Natural History, New York, NY, USA; USNM - National Museum of Natural History, Smithsonian Institution, Division of Mammals, Washington, D.C., USA; NMNHP, Primate Collection of the Department of Comparative Anatomy of the National Museum of Natural History, Paris, France; Gombe - the primate collection of the University of Minnesota from the Gombe Chimpanzee Research Project, Minneapolis, MN, USA; Tervuren - the Royal Museum for Central Africa, Tervuren, Belgium; DAUC - Duckworth Laboratory, Department of Archaeology, University of Cambridge, UK.

Table S9. Composition of the living and archaeological *H. sapiens* sample.

**Population**	**Chronology**	**Subsistence**	**Sex**	**Total**	**Institution/ Collection**[b]
**Living *H. sapiens***					
Control (well-developed longitudinal arch)	21st century	urban labors and specializations	10Fs, 5Ms	15	UCSM
Congenital flexible flatfoot	21st century	urban labors and specializations	3Fs, 3Ms	6	UCSM
Adult acquired flexible flatfoot	21st century	urban labors and specializations	4Fs, 1M	5	IOR
**Archaeological *H. sapiens***					
Bologna (Italy)	20th century	agrarian, maritime, urban labors and specializations	24Fs, 25 Ms	49	UniBO - BiGeA, Certosa Collection
Sassari (Italy)	20th century	agrarian, maritime, urban labors and specializations	20 Fs, 22Ms	42	UniBO - BiGeA Frassetto Collection
South Africa	19th-20th centuries	urban labors and specializations	4Fs, 13Ms	17	Dart Collection
Via Orfeo, Bologna (Italy; Jewish Cemetery)	14th-16th century	urban (bankers, traders, artisans)	10Fs, 14Ms	24	UniBO - BiGeA
Roccapelago, Modena (Italy)	17th-18th century	Herders, mountain dwellers	24U	24	UniBO - DBC
Suasa, Ancona (Italy)	1st -3rd century	Farmers	9Fs, 10Ms, 4U	23	UniBO - BiGeA
Olmo di Nogara, Verona (Italy)	Bronze Age, 1750-1300 BC	Herders, agriculturalists	5U	5	UniPI
Beli Manastir (Croatia)	Eneolithic, 3000-2200 BC	Herder, agriculturalist	1M	1	IAR
Osijek, Beli Manastir (Croatian sites)	Early/Middle Neolithic, 6000-5000 BC	Agro-pastoralists	3Fs, 6Ms	9	IAR
Al Khiday (Central-Sudan)	Middle Mesolithic, 7050 - 6400 cal BC	Hunter–gatherers and fishing	10U	10	UniPD Al-Khiday collection
Bloemfontein, Iziko (South Africa)	Late Stone Age, 11250 - 3040 BP	Hunter-gatherers	2Fs, 5Ms, 18U	25	NMB

UCSM - University of Colorado School of Medicine, USA; IOR - Istituto Ortopedico Rizzoli, Italy; UniBO – BiGeA - University of Bologna, Department of Biological, Geological and Environmental Sciences, Italy; UniBO – DBC - University of Bologna, Department of Cultural Heritage, Italy; Dart Collection - University of the Witwatersrand, Johannesburg, South Africa; UniPI - University of Pisa, Department of Biology, Italy; IAR - Centre for Applied Bioanthropology, Institute for Anthropological Research, Zagreb, Croatia; UniPD - Università degli Studi di Padova, Museo di Antropologia, Italy; NMB - Florisband Quaternary Research Station of the National Museum of Bloemfontein, South Africa. F – female; M – male; U – unknown.

Table S10. Fossil sample.

Fossils	Taxonomy	Age	Institution/Collection	References
Dolní Věstonice 15	Upper Pleistocene *Homo sapiens*	~31 ka cal BP	Institute of Archeology Brno	Fewlass et al. 2019[1]; Formicola et al., 2001[2]
Dolní Věstonice 16	Upper Pleistocene *Homo sapiens*	~31 ka cal BP	Institute of Archeology Brno	Fewlass et al. 2019[1]; Formicola et al., 2001[2]
Qafzeh 8	late Middle Pleistocene/ early Upper Pleistocene *Homo sapiens*	~92ka - <115 ka	University Tel Aviv	Schwarcz et al. 1988[3]; Vandermeersch & Bar-Yosef, 2019[4]
Qafzeh 9	late Middle Pleistocene/ early Upper Pleistocene *Homo sapiens*	~92ka - <115 ka	University Tel Aviv	Schwarcz et al. 1988[3]; Vandermeersch & Bar-Yosef, 2019[4]
Skhul 4	late Middle Pleistocene/ early Upper Pleistocene *Homo sapiens*	~100-130 ka	Rockefeller Museum Jerusalem	Grün et al. 2005[5]
KHS 1-45 (Omo-Kibish 1)	late Middle Pleistocene/ early Upper *Pleistocene Homo sapiens*	195 ka	Ethiopian Authority for Research and Conservation of Cultural Heritage	Pearson et al., 2008[6]; DeSilva et al., 2019[7]
La Ferrassie 1	*H. neanderthalensis*	40 - 54 ka	National History Museum Paris, Anthropology Collection	Guérin,et al. 2015[8]
La Ferrassie 2	*H. neanderthalensis*	40 - 54 ka	National History Museum Paris, Anthropology Collection	Guérin,et al. 2015[8]
LB1/16	*Homo floresiensis*	60 ka	The National Research Centre for Archaeology, Indonesia	Jungers et al., 2009[9]; Prang, 2016[10]; DeSilva et al., 2019[7]
U.W. 101-1758	*Homo naledi*	240–340 ka	University of the Witwatersrand	Harcourt-Smith et al., 2015[11]; DeSilva et al., 2019[7]
OH 8	*Homo habilis/ Paranthropus boisei?*	1.85 Ma	National Museum of Tanzania	Sarmiento & Marcus, 2000[12]; Prang, 2016; DeSilva et al., 2019
A.L. 333-36	*Australopithecus afarensis*	3.2 Ma	Ethiopian Authority for Research and Conservation of Cultural Heritage	Sarmiento & Marcus, 2000[1]; Prang, 2016[7]; DeSilva et al., 2019[7]
A.L. 333-47	*Australopithecus afarensis*	3.2 Ma	Ethiopian Authority for Research and Conservation of Cultural Heritage	Sarmiento & Marcus, 2000[1]; Prang, 2016[7]; DeSilva et al., 2019[7]
StW 573	*Australopithecus prometheus*	3.67 Ma	University of the Witwatersrand	Clarke & Tobias, 1995[13]; Granger et al., 2015[14]; DeSilva et al., 2019[7]

Table S11. Landmarks and semi-landmarks of the navicular template.

Landmarks	Definition
**1**	Most medial point of the talar facet
**2**	Most plantar point of margin separating medial and intermediate cuneiform facets
**3**	Most plantar point of margin separating lateral and intermediate cuneiform facets
**4**	Most dorsal point of margin separating lateral and intermediate cuneiform facets
**5**	Most dorsal point of margin separating medial and intermediate cuneiform facets

Curves	Definition	Number of semi-landmarks
**1->1**	Outline of the talar facet	19
**2->3**	Plantar margin of the intermediate cuneiform facet	1
**2->5**	Dorsal, medial, and plantar margins of the medial cuneiform facet	9
**5->2**	Margin separating medial and intermediate cuneiform facets	3
**5->4**	Dorsal margin of the intermediate cuneiform facet	4
**4->3**	Margin separating lateral and intermediate cuneiform facets	3
**3->4**	Dorsal, lateral, and plantar margins of the lateral cuneiform facet	7

Surfaces	Number of semi-landmarks
**Talar facet**	11
**Medial cuneiform facet**	5
**Intermediate cuneiform facet**	3
**Lateral cuneiform facet**	3
**Tuberosity (non-articular surface)**	12

Supplementary References

1. Fewlass, H. *et al.* Direct radiocarbon dates of mid Upper Palaeolithic human remains from Dolní Věstonice II and Pavlov I, Czech Republic. *J. Archaeol. Sci. Reports* **27**, 102000 (2019).
2. Formicola, V., Pontrandolfi, A. & Svoboda, J. The upper paleolithic triple burial of Dolní Věstonice: Pathology and funerary behavior. Am. J. Phys. Anthropol. **115**, 372–379 (2001).
3. Schwarcz, H. P., *et al.* ESR dates for the hominid burial site of Qafzeh in Israel. *J. Hum. Evol.* **17**, 733-737 (1988).
4. Vandermeersch, B., & Bar-Yosef, O. The paleolithic burials at Qafzeh cave, Israel. *PALEO. Revue d'archéologie préhistorique* **30-1**, 256-275 (2019).
5. Grün, R., *et al.* U-series and ESR analyses of bones and teeth relating to the human burials from Skhul. *J. Hum. Evol.* **49**, 316-334 (2005).
6. Pearson, O. M., Royer, D. F., Grine, F. E., & Fleagle, J. G. A description of the Omo I postcranial skeleton, including newly discovered fossils. *J. Hum. Evol.* **55**, 421-437 (2008).
7. DeSilva, J., McNutt, E., Benoit, J. & Zipfel, B. One small step: A review of Plio-Pleistocene hominin foot evolution. *Am. J. Phys. Anthropol.* **168**, 63–140 (2019).
8. Guérin, G., *et al.* A multi-method luminescence dating of the Palaeolithic sequence of La Ferrassie based on new excavations adjacent to the La Ferrassie 1 and 2 skeletons. *J. Archaeol. Sci.* **58**, 147-166 (2015).
9. Jungers, W. L. *et al.* The foot of Homo floresiensis. *Nature* **459**, 81–84 (2009).
10. Prang, T. C. Reevaluating the functional implications of *Australopithecus afarensis* navicular morphology. *J. Hum. Evol.* **97**, 73–85 (2016).
11. Harcourt-Smith, W. H. E. *et al.* The foot of Homo naledi. *Nat. Commun.* **6**, 1–8 (2015).
12. Sarmiento, E. E. & Marcus, L. F. The os navicular of humans, great apes, OH 8, Hadar, and Oreopithecus: Function, phylogeny, and multivariate analyses. *Am. Museum Novit.* **2000**, 1–38 (2000).
13. Clarke, R. J. & Tobias, P.V. Sterkfontein Member 2 foot bones of the oldest South African hominid. *Science* **269**, 521-524 (1995).
14. Granger, D. E., *et al.* New cosmogenic burial ages for Sterkfontein Member 2 Australopithecus and Member 5 Oldowan. *Nature* **522**, 85-88 (2015).
