# Supplementary material for: Morphological and evolutionary insights into the keystone element of the human foot’s medial longitudinal arch
Source: Commun Biol. 2023 Oct 19;6:1061. doi: 10.1038/s42003-023-05431-8 (PMC10587292; doi:10.1038/s42003-023-05431-8)
Supplement: Supplementary file 2 — Description of Additional Supplementary Files [file 42003_2023_5431_MOESM2_ESM.pdf]

Description of Additional Supplementary Files

File name: Supplementary Data 1
Description: This file contains –

1. A file provides coordinates (X, Y, Z) extracted from Viewbox for each landmark and semi landmark used in the study.
2. The full sample list.
3. The centroid size of the full sample.
4. The list of landmarks and semi landmarks on curve used to compute Generalised Procrustes.
5. The file provides the list of sliding semi landmarks used for Generalised Procrustes Analysis.
