# Supplementary material for: Morphological and evolutionary insights into the keystone element of the human foot’s medial longitudinal arch
Source: Commun Biol. 2023 Oct 19;6:1061. doi: 10.1038/s42003-023-05431-8 (PMC10587292; doi:10.1038/s42003-023-05431-8)
Supplement: Supplementary file 4 — Reporting Summary [file 42003_2023_5431_MOESM4_ESM.pdf]

nature portfolio

Corresponding author(s): Rita Sorrentino

Last updated by author(s): Sep 27, 2023

Reporting Summary

Nature Portfolio wishes to improve the reproducibility of the work that we publish. This form provides structure for consistency and transparency in reporting. For further information on Nature Portfolio policies, see our Editorial Policies and the Editorial Policy Checklist.

Statistics

For all statistical analyses, confirm that the following items are present in the figure legend, table legend, main text, or Methods section.

n/a	Confirmed	
☐	☒	The exact sample size (*n*) for each experimental group/condition, given as a discrete number and unit of measurement
☐	☒	A statement on whether measurements were taken from distinct samples or whether the same sample was measured repeatedly
☐	☒	The statistical test(s) used AND whether they are one- or two-sided<br>*Only common tests should be described solely by name; describe more complex techniques in the Methods section.*
☐	☒	A description of all covariates tested
☐	☒	A description of any assumptions or corrections, such as tests of normality and adjustment for multiple comparisons
☐	☒	A full description of the statistical parameters including central tendency (e.g. means) or other basic estimates (e.g. regression coefficient) AND variation (e.g. standard deviation) or associated estimates of uncertainty (e.g. confidence intervals)
☐	☒	For null hypothesis testing, the test statistic (e.g. *F*, *t*, *r*) with confidence intervals, effect sizes, degrees of freedom and *P* value noted<br>*Give P values as exact values whenever suitable.*
☒	☐	For Bayesian analysis, information on the choice of priors and Markov chain Monte Carlo settings
☒	☐	For hierarchical and complex designs, identification of the appropriate level for tests and full reporting of outcomes
☐	☒	Estimates of effect sizes (e.g. Cohen's *d*, Pearson's *r*), indicating how they were calculated

Our web collection on statistics for biologists contains articles on many of the points above.

Software and code

Policy information about availability of computer code

Data collection	Data (navicular bones of living and fossil subjects, as described in the manuscript) were collected by laser or blue light scanning, by computed tomography (CT), and by micro CT.
Data analysis	For data analysis of CT and micro CT scans, we used the licensed software Avizo v. 9.2 (Thermo Fisher Scientific, Waltham). Creation and application of the geometric morphometric template were made in the licensed software Viewbox v. 4 (dHAL software, Kifissia). Statistical analyses were conducted in the R version 4.0.5 using freeware R packages as described in the manuscript.

For manuscripts utilizing custom algorithms or software that are central to the research but not yet described in published literature, software must be made available to editors and reviewers. We strongly encourage code deposition in a community repository (e.g. GitHub). See the Nature Portfolio guidelines for submitting code & software for further information.

Data

Policy information about availability of data

All manuscripts must include a data availability statement. This statement should provide the following information, where applicable:

- Accession codes, unique identifiers, or web links for publicly available datasets
- A description of any restrictions on data availability
- For clinical datasets or third party data, please ensure that the statement adheres to our policy

All data needed to evaluate the conclusions in the paper are present in the manuscript and/or the Supplementary Information. Digital models of non-human apes

from the AMNH and USNM are available from www.MorphoSource.org (Anthropoid Primate Feet). Other 3D models of naviculars are available from the authors; however, restrictions apply to the availability of these data since they were used explicitly under license for the current study and are not publicly accessible. The 3D geometric morphometric data (i.e., landmarks coordinates) are available in the Supplementary Data 2File.

Human research participants

Policy information about studies involving human research participants and Sex and Gender in Research.

Reporting on sex and gender	Analyses based on sex and gender were not performed, as this topic is beyond the research question of this manuscript. The biological sex of the sample is given in Supplementary Tables 8 and 9.
Population characteristics	CT scans of the foot bones of a living Homo sapiens sample (older than 18 years) were selected after an assessment of foot type by a clinician (described in the Data Collection section of the manuscript).
Recruitment	Control and congenital flexible flatfoot specimens were acquired by cone beam CT in weight-bearing conditions (CurveBeam, Philadelphia, USA) at the Department of Orthopedics, School of Medicine, University of Colorado, USA. Five adult flexible flat foot scans were acquired by cone beam CT in weight-bearing ('OnSight 3D Extremity System', Carestream, Rochester, NY) at IRCCS Istituto Ortopedico Rizzoli - IOR, Italy.
Ethics oversight	Scans of control and congenital flexible flatfoot specimens were originally obtained for standard clinical care purposes, and then retrospectively collected and selected for use in this study after approval by the Colorado Multiple Institutional Review Board. CT scans of five adult flexible flatfoot, acquired at IRCCS Istituto Ortopedico Rizzoli, were performed in accordance with relevant national guidelines and regulations, and informed consent was obtained from these five patients.

Note that full information on the approval of the study protocol must also be provided in the manuscript.

Field-specific reporting

Please select the one below that is the best fit for your research. If you are not sure, read the appropriate sections before making your selection.

☐ Life sciences ☐ Behavioural & social sciences ☒ Ecological, evolutionary & environmental sciences

For a reference copy of the document with all sections, see nature.com/documents/nr-reporting-summary-flat.pdf

Ecological, evolutionary & environmental sciences study design

All studies must disclose on these points even when the disclosure is negative.

Study description	This study analyzes the external morphology of the navicular bone of 357 extant and 14 extinct hominid navicles (Table S8 and S9). Considering that the navicular bone is the keystone of the medial longitudinal arch of Homo sapiens due to its position in the midfoot, this study aims to evaluate the navicular morphology of hominids and assess its anatomical correlates with the medial longitudinal arch.
Research sample	The study sample includes bones from H. sapiens (living, archaeological, and fossil), great apes, and fossil hominins as enumerated in Table S8, S9, and S10.
Sampling strategy	Extant sample sizes were based on the availability of 3D models or digital acquisition of adult human and (non-human) great ape naviculars in Institution or Museum collections as reported in Table S8 and S9. Fossil sample sizes were based on the number of specimens for which the authors had permissions to analyze.
Data collection	R.S., C.M.O., A.P., C.F., S.L., M.C., N.S., C.B., M.Z., L.F., J.J.H., T.J., M.N., B.A.P., T.C.P., J.P.P.S., M.M., A.L., J.D. collected the scan data
Timing and spatial scale	Some of the data collection began in 2018, while most was conducted between 2020-2022.
Data exclusions	After a GPA, a total of 9 individuals from the Homo sapiens sample was detected as outliers and, consequently, they were removed from the final analysis: one male individual from Bologna, two male individuals from Sotho, one individual from Roccapelago (unknown sex), two individuals from Via Orfeo (one male and one female), three individuals from Al Khiday (unknown sex).
Reproducibility	The 3D surface renderings were acquired through laser or blue light scanning, computed tomography (CT) and micro-CT scanning. Regardless of the triangulation size defining surface, all digital techniques give comparable results and can be used in the same analysis.
Randomization	The compared specimens have a known taxonomic allocation. The affinity of fossil hominins to extant taxa was calculated through a classification test on the probability that each fossil hominin belongs to an extant taxon, based on Mahalanobis distance (D2). Shape differences between archaeological and living Homo sapiens groups were tested through the permutation test (1,000).
Blinding	Blinding was not necessary in this study as the data has taxonomical allocation.

Did the study involve field work? ☐ Yes ☒ No

Reporting for specific materials, systems and methods

We require information from authors about some types of materials, experimental systems and methods used in many studies. Here, indicate whether each material, system or method listed is relevant to your study. If you are not sure if a list item applies to your research, read the appropriate section before selecting a response.

Materials & experimental systems

n/a	Involved in the study
☒	☐ Antibodies
☒	☐ Eukaryotic cell lines
☒	☐ Palaeontology and archaeology
☒	☐ Animals and other organisms
☒	☐ Clinical data
☒	☐ Dual use research of concern

Methods

n/a	Involved in the study
☒	☐ ChIP-seq
☒	☐ Flow cytometry
☒	☐ MRI-based neuroimaging
